# Bulk-Surface Electrothermodynamics and Applications to Electrochemistry

**DOI:** 10.3390/e20120939

**Published:** 2018-12-06

**Authors:** Wolfgang Dreyer, Clemens Guhlke, Rüdiger Müller

**Affiliations:** Weierstrass-Institute, Mohrenstr. 39, 10117 Berlin, Germany

**Keywords:** bulk-surface electrothermodynamics, entropy principle, constitutive modeling, electrochemistry

## Abstract

We propose a modeling framework for magnetizable, polarizable, elastic, viscous, heat conducting, reactive mixtures in contact with interfaces. To this end, we first introduce bulk and surface balance equations that contain several constitutive quantities. For further modeling of the constitutive quantities, we formulate constitutive principles. They are based on an axiomatic introduction of the entropy principle and the postulation of Galilean symmetry. We apply the proposed formalism to derive constitutive relations in a rather abstract setting. For illustration of the developed procedure, we state an explicit isothermal material model for liquid electrolyte|metal electrode interfaces in terms of free energy densities in the bulk and on the surface. Finally, we give a survey of recent advancements in the understanding of electrochemical interfaces that were based on this model.

## 1. Introduction

The energy transition from fossil fuels to renewable energy sources gives rise to an increasing demand for more efficient energy storage in stationary and mobile applications, cf. [1]. As current lithium-ion battery technology is expected to reach its theoretical limit soon, several promising future technologies like metal-sulfur and metal-air systems, as well as polymer electrolyte batteries and different other types of solid state batteries, are intensively investigated [2,3,4,5].

Because the demand for both portable water and clean water for industry and agriculture is expected to grow, there is a strong need for better water desalination and water purification technologies. Electrically driven processes like electrodialysis and capacitive deionization are investigated since they promise to be commercially competitive and in particular energy efficient, compared to thermal or pressure driven processes, [6,7,8].

A key ingredient for better understanding of the electrochemical processes in the mentioned technologies is the development of better mathematical models. Standard models like the Poisson–Nernst–Planck system, cf. e.g., [9,10], suffer from deficiencies that are well known. Within a continuum theory, some of these flaws have already been remedied, see e.g., [11,12] and the literature cited therein. However, to develop models for the above mentioned applications, there is the more fundamental problem that additional effects like elastic deformation, stresses or interaction of charge transport with fluid flow have to be included into the models. However, extension of the standard model to cover these further effects and other material properties in a consistent way are far from being obvious.

The aim of this work is the formulation of a general modeling framework that allows the derivation of strictly thermodynamically consistent models for the above applications. With this article, we want to provide a revised and more concise version of [13] and want to make it available to a broader audience. Thereby, we close a gab that remained in our recent works on the improved continuum modeling of electrochemical interfaces [14,15,16,17,18,19]. We here resume the effort of non-equilibrium thermodynamics containing electromagnetic fields and its extensions to surfaces [20,21,22,23], which was combined to a single unified framework in [13] for the first time, together with the derivation of the according constitutive equations. Extending [24] to electromagnetic fields and to surfaces, we build our model on (i) universally valid balance equations in the bulk and on surfaces on the one hand and (ii) the formulation of an entropy principle and the according derivation of constitutive relations on the other hand. In particular, a chemically reacting mixture with neutral and electrically charged components will be described. Other phenomena such as diffusion, heat conduction, viscosity, elasticity, polarization and magnetization are taken into account.

When applying the resulting continuum models to electrochemical systems, typically very different scales in space and time arise and not all of them are necessary to capture the macroscopic relevant features of the considered system. Here, we show by means of dimensional analysis how considerable simplification of the model is possible and still—compared to the standard literature—a much more fundamental approach like [14] is possible to overcome deficiencies of the classical Nernst–Planck model. Thereby, we gain better insight into the internal double layer structure and the electrolyte transport by diffusive fluxes. Moreover, asymptotic analysis provides a mathematical tool to derive more simple reduced models. By this procedure, it is possible to recover on a macroscopic level the double layer capacity of electrode-aqueous electrolyte interfaces and some well established relations of electrochemistry, like Butler–Volmer equations and the Lippmann equation.

### Outline

This paper is organized as follows: in the following section, we introduce notation and the geometrical setup. Then, in Section 3, we state the universal balance equations in the bulk and on the surface, containing the balances for the fields of matter and Maxwell’s equations and their coupling. The transformation properties of the involved fields and the principle of Galilean symmetry are briefly discussed and stated in Section 4. In Section 5, we formulate the axioms of the entropy principle in bulk and surface and describe the general closing procedure based on the exploitation of the entropy principle. Subsequently, we derive in Section 6 constitutive relations in bulk and surfaces for magnetizable, polarizable, viscous and reactive mixtures and discuss in particular the role of polarization and magnetization. In Section 7, we apply the general model to liquid electrolytes, in particular aqueous electrolytes in contact with metal electodes and give a survey over recent key results. We close with some concluding remarks in Section 8.

## 2. General Setting and Basic Quantities

### 2.1. Geometrical Setup

**Figure 1:**
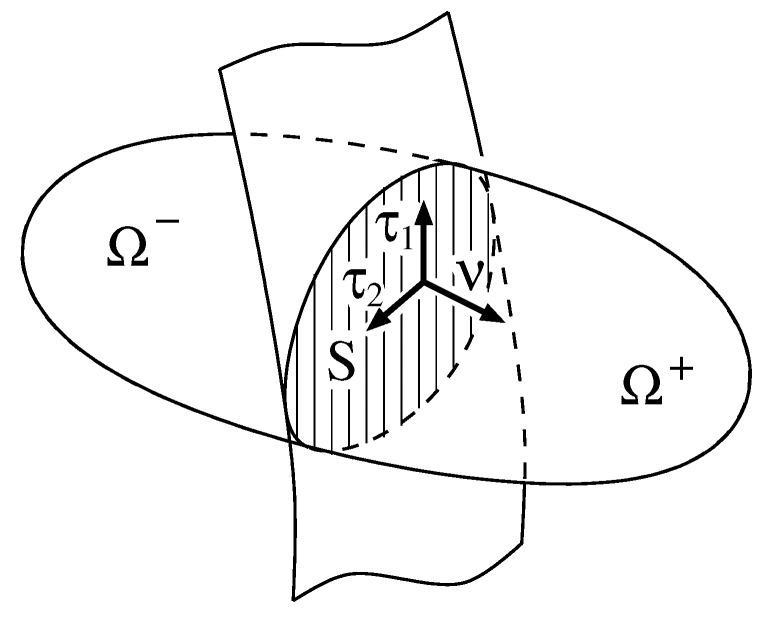
A surface *S* separating a domain Ω into two subdomains $\Omega^{\pm }$.

We consider a moving orientable surface S(t) dividing a possibly evolving domain $\Omega \left(t\right)\subset R^{3}$ into two subdomains $\Omega^{\pm }\left(t\right)\subseteq R^{3}$ with $S:=\partial \Omega^{+}\cap \partial \Omega^{-}$—see Figure 1. Let $\theta :[0,t_{\mathrm{end}})\times \omega \to S$ be a smooth bijective parametrization of the surface *S*, where $\omega \subset R^{2}$ is the open parameter domain. The partial derivatives of θ define tangential vectors, the surface normal and the metric

(1) $$\tau_{1/2}=\frac{\partial \theta (t,U^{1},U^{2})}{\partial U^{1/2}}\;,\;\nu =\frac{\tau_{1}\times \tau_{2}}{|\tau_{1}\times \tau_{2}|}\;\;\mathrm{and}\;\;g={\left[\tau_{1},\tau_{2}\right]}^{T}\left[\tau_{1},\tau_{2}\right]\;.$$

As a convention, we chose the orientation of the surface normal such that ν is the inner normal of $\Omega^{+}$. Moreover, we indicate the components of vectors and tensors with respect to Cartesian coordinates by lowercase Latin indices, e.g., i,j,k, whereas we use uppercase Greek indices like e.g., Γ,Δ,Σ for the tangential components. For a vector V defined on the surface, we denote the normal component by $V_{\nu }$ and write $V_{\tau }^{\Delta }$, for Δ=1,2, for the tangential components. For the matrix components of the metric tensor g, we use lower indices $g_{\Delta \Gamma }$ and, for the components of the inverse matrix of the metric, we use upper indices $g^{\Delta \Gamma }$. We apply the convention of implicit summation over coordinate indices appearing twice.

The *curvature tensor* $b_{\Delta \Gamma }$ of the surface *S* and the *Christoffel symbols* $\Gamma_{\Delta \Gamma }^{\Sigma }$ are defined by a decomposition of the derivatives of the tangential vectors into their tangential and normal components,

(2) $$\frac{\partial \tau_{\Delta }}{\partial U^{\Gamma }}=\Gamma_{\Delta \Gamma }^{\Sigma }\tau_{\Sigma }+b_{\Delta \Gamma }\;\nu \;\;\mathrm{for}\;\Gamma ,\Delta =1,2\;.$$

Then, the mean curvature of *S* is $k_{M}=\frac{1}{2}b_{\Gamma \Delta }g^{\Gamma \Delta }$.

#### Covariant Derivatives

Let a:S→R be a scalar and $V:S\to R^{3}$ a vector field. Then, the covariant derivatives of the tangential components are defined as

(3) $$\begin{array}{cc} a_{∥\Gamma }=\frac{\partial a}{\partial U^{\Gamma }}\;,\;\;\mathrm{for}\;\Gamma =1,2\;, & V_{\tau ∥\Gamma }^{\Delta }=\frac{\partial V_{\tau }^{\Delta }}{\partial U^{\Gamma }}+\Gamma_{\Gamma \Sigma }^{\Delta }V_{\tau }^{\Sigma }\;\;\mathrm{for}\;\Gamma ,\Delta =1,2\;. \end{array}$$

Let w denote the velocity of the surface *S*. For a scalar $a:[0,t_{\mathrm{end}})\times S\to R$, we define the time derivative

(4) $$\partial_{t,\nu }a=\partial_{t}a-a_{∥\Delta }\;w_{\tau }^{\Delta }\;.$$

#### Traces, Jumps and Mean Values on Surfaces

For a generic function $u:\left[0,t_{\mathrm{end}}\right)\times \left(\Omega^{+}\cup \Omega^{-}\right)\to R^{m},$ we denote for any time *t* and for each $\underset{s}{x}\;\;\in S$ the *trace* on either side of the surface by

(5) $$u^{\pm }\left(t,\underset{s}{x}\;\;\right)=\lim_{x\in \Omega^{\pm }\to \underset{s}{x}\;\;\in S}u\left(t,x\right)\;.$$

If the function *u* is not defined in $\Omega^{+}$ or $\Omega^{-}$, we set the corresponding trace to zero, i.e., $u^{+}=0$ if *u* is not defined in $\Omega^{+}$ and $u^{-}=0$ if *u* is not defined in $\Omega^{-}$. By

(6) $$\begin{array}{cc} \left[\;[u]\;\right]=u^{+}-u^{-}\;, & \bar{u}=\frac{1}{2}\left(u^{+}+u^{-}\right), \end{array}$$

we define the *jump* and the *mean value* of *u* at the surface *S*, respectively. The definition of the jump is related to the above convention on the orientation of the surface normal to be the inner normal of $\Omega^{+}$, cf. Figure 1.

### 2.2. Description of Reacting Mixtures

For quantities defined in the domains $\Omega^{+}$ or $\Omega^{-}$, there will often be corresponding quantities on the surfaces *S*. As a convention, the same letters are used for these quantities, but the surface variables are indicated by an underset *s*.

#### Constituents and Chemical Reactions

In each of the two domains $\Omega^{+}$ and $\Omega^{-}$ and on the surface *S*, we consider a mixture of several constituents, usually referenced as $A_{\alpha }$. The index set of constituents in $\Omega^{\pm }$ is denoted by $I^{\pm }$. We assume that the sets $I^{\pm }$ are disjoint, i.e., $I^{+}\cap I^{-}=\emptyset$. This assumption takes into account that, even if a certain chemical substance is present in both domains, the functional dependence of the corresponding chemical potentials in general differs between the subdomains. All constituents of the subdomains $\Omega^{\pm }$ are assumed to be also constituents on the surface *S*, but, in addition, there may be some constituents that are exclusively present on *S*. Accordingly, the constituents on the surface *S* are represented by the set $I_{S}$ with $I^{\pm }\subseteq I_{S}$. For the later construction of constitutive equations, we choose in each subdomain one designated constituent as a reference. We denote these constituents by $A_{0-}$, $A_{0+}$, $A_{0}$ in $\Omega^{-}$, $\Omega^{+}$ and *S*, respectively.

For a description on a continuum level (macroscopic level), each of constituent $A_{\alpha }$ is characterized by the (atomic) mass $m_{\alpha }$ and its atomic net charge $z_{\alpha }e_{0}$, where the positive constant $e_{0}$ is the elementary charge and $z_{\alpha }$ is the charge number of the constituent. There may be M≥0 chemical reactions among the bulk constituents and $M_{S}\geq 0$ chemical reactions on the surface. These reactions may be written in the general form

(7a) $$\sum_{\alpha \in I^{\pm }}a_{\alpha }^{k}A_{\alpha }\overset{R_{k}^{b}}{\underset{R_{k}^{f}}{⇌}}\sum_{\alpha \in I^{\pm }}b_{\alpha }^{k}A_{\alpha }\;\mathrm{for}\;k\in \{1,\cdots ,M\}\;,$$

(7b) $$\sum_{\alpha \in I_{S}}\underset{s}{a}\;{\;}_{\alpha }^{k}A_{\alpha }\overset{\underset{s}{R_{k}^{b}}}{\underset{\underset{s}{R_{k}^{f}}}{⇌}}\sum_{\alpha \in I_{S}}\underset{s}{b}\;{\;}_{\alpha }^{k}A_{\alpha }\;\mathrm{for}\;k\in \{1,\cdots ,M_{S}\}\;.$$

The constants $a_{\alpha }^{k}$, $b_{\alpha }^{k}$ are positive integers and $\gamma_{\alpha }^{k}:=b_{\alpha }^{k}-a_{\alpha }^{k}$ denote the stoichiometric coefficients of the reactions. The reaction from left to right is called forward reaction with reaction rate $R_{f}^{k}>0$. The reaction in the reverse direction with rate $R_{b}^{k}>0$ is the backward reaction. The net reaction rate is defined as $R^{k}=R_{f}^{k}-R_{b}^{k}$. Since charge and mass have to be conserved by each single reaction in the bulk and on the surface, we have

(8a) $$\sum_{\alpha \in I^{\pm }}z_{\alpha }\gamma_{\alpha }^{k}=0\;\mathrm{and}\;\sum_{\alpha \in I^{\pm }}m_{\alpha }\gamma_{\alpha }^{k}=0\;\;\mathrm{for}\;k\in \{1,\cdots ,M\}\;,$$

(8b) $$\sum_{\alpha \in I_{S}}z_{\alpha }\underset{s}{\gamma_{\alpha }^{k}}\;\;=0\;\mathrm{and}\;\sum_{\alpha \in I_{S}}m_{\alpha }\underset{s}{\gamma_{\alpha }^{k}}\;\;=0\;\;\mathrm{for}\;k\in \{1,\cdots ,M_{S}\}\;.$$

#### Electro- and Thermodynamic State

The thermodynamic state of $\Omega^{\pm }$ at any time *t* is described by the number densities $n_{\alpha }$ and the velocities $\upsilon_{\alpha }$ for $\alpha \in I^{\pm }$ and the (specific) internal energy *u*. Analogously, the thermodynamic state of the surface *S* is characterized by the number densities of the surface constituents $\underset{s}{n}\;{\;}_{\alpha }$ and the surface velocities $\underset{s}{\upsilon }\;{\;}_{\alpha }$ for $\alpha \in I_{S}$ and the (specific) surface internal energy $\underset{s}{u}\;\;$. The electrodynamic state of $\Omega^{\pm }$ and surface *S* at any time *t* is described by the electric field E and the magnetic field B. Alternatively, the electromagnetic behavior of polarizable and magnetizable matter could equally well be characterized by the vectors of polarization P and magnetization M, instead of E and B. Both sets of variables, (E,B) and (P,M), are coupled by constitutive equations and, for the derivation of these relations, it turns out to be favourable to use P and M, for reasons which are discussed in Section 6.4.

Multiplication of the number densities $n_{\alpha }$ by $m_{\alpha }$ gives the partial mass densities

(9) $$\rho_{\alpha }=m_{\alpha }n_{\alpha }\;,\;\underset{s}{\rho }\;{\;}_{\alpha }=m_{\alpha }\underset{s}{n}\;{\;}_{\alpha }\;.$$

The mass density and the barycentric velocity of the mixture are defined by

(10a) $$\begin{array}{cccc} \rho & =\sum_{\alpha \in I^{\pm }}\rho_{\alpha } & \;\mathrm{and}\;\;\upsilon & =\frac{1}{\rho }\sum_{\alpha \in I^{\pm }}\rho_{\alpha }\upsilon_{\alpha }\;, \end{array}$$

(10b) $$\begin{array}{cccc} \underset{s}{\rho }\;\; & =\sum_{\alpha \in I_{S}}\underset{s}{\rho }\;{\;}_{\alpha } & \;\mathrm{and}\;\;\underset{s}{\upsilon }\;\; & =\frac{1}{\underset{s}{\rho }\;\;}\sum_{\alpha \in I_{S}}\underset{s}{\rho }\;{\;}_{\alpha }\underset{s}{\upsilon }\;{\;}_{\alpha }\;. \end{array}$$

The free charge density is defined as

(11) $$\begin{array}{cccc} n^{F} & =\sum_{\alpha \in I^{\pm }}z_{\alpha }e_{0}n_{\alpha }\;, & \underset{s}{n}\;{\;}^{F} & =\sum_{\alpha \in I_{S}}z_{\alpha }e_{0}\underset{s}{n}\;{\;}_{\alpha }\;. \end{array}$$

The internal electronic structure of the constituents is not reflected by our macroscopic description, but it has relevance for the overall electromagnetic field. To represent these microscopic effects on the more macroscopic level, we introduce the *polarization charge density* $n^{P}$, $\underset{s}{n}\;{\;}^{P}$ in $\Omega^{\pm }$ and *S*, respectively, and then define the total charge density by

(12) $$\begin{array}{cccc} n^{e} & =n^{F}+n^{P}\;, & \underset{s}{n}\;{\;}^{e} & =\underset{s}{n}\;{\;}^{F}+\underset{s}{n}\;{\;}^{P}\;. \end{array}$$

#### Deformation Gradient

The barycentric velocity causes deformation of bulk and surface. We formally introduce the deformation gradient F by means of a partial differential equation, viz.

(13) $$\frac{\partial F}{\partial t}+\left(\upsilon \cdot \nabla \right)F=F^{T}\left(\nabla \upsilon \right)\;,$$

where det(F)>0 is assumed. For the relation of the so defined F to the deformation of a body under the velocity υ, we refer to the textbook literature, cf. e.g., ([25] p. 328, Equation (67.4)). From the deformation gradient, the unimodular, or volume preseving, deformation gradient $F^{\mathrm{uni}}$ is derived as

(14) $$F^{\mathrm{uni}}=\det{\left(F\right)}^{-\frac{1}{3}}F\;\mathrm{implying}\;\;\det\left(F^{\mathrm{uni}}\right)=1\;.$$

The temporal changes of the surface parametrization θ describes the deformation of the surface. Therefore, the tangential vectors $\tau_{1/2}$ are the surface equivalents of the deformation gradients. Similar to the bulk, the unimodular tangential vectors are defined as

(15) $$\tau_{1/2}^{\mathrm{uni}}=\det{\left(g\right)}^{-\frac{1}{4}}\tau_{1/2}\;\mathrm{implying}\;\;\det\left(\left[\tau_{1}^{\mathrm{uni}},\tau_{2}^{\mathrm{uni}}\right]\right)=1\;.$$

## 3. Universal Balance Equations of Electrothermodynamics

In this section, we introduce a set of balance equations in the bulk and on the surface that we postulate to hold universally, i.e., these balances are assumed to hold independent from the considered material. The surface balances account for the transport in tangential as well as in normal direction. Our approach is oriented on the classical work of Truesdell and Toupin [25] and we apply a notation similar to [21]. In this work, we consider only local balance equations that can be derived from their respective global counterparts, cf. [21].

The set of balance equations is subdivided into the classical balances of matter on the one hand and Maxwell’s equations for the electromagnetic field on the other hand. Following [25], Maxwell’s equations in the bulk are formulated with the postulation of universally valid Maxwell–Lorentz aether relations. For the surface, there is not such a standard formulation of the equations and, in particular, we here derive surface equations by analogy to the procedure in the bulk. With the later application to electrochemical systems in mind, we use the classical, i.e., non-relativistic form of the balances for the fields of matter.

### 3.1. Balance Equations of Matter

We consider partial mass balances for each of the constituents of the mixture, a single momentum balance of the mixture and an energy balance of matter. Modeling approaches based on different sets of balance equations also exists. For instance, each partial momentum $\rho_{\alpha }\upsilon_{\alpha }$ can be balanced. An introduction to these kinds of models for the bulk systems without surface balances and without electrodynamics can be found in [24].

#### Balance of Mass

In each of the subdomains $\Omega^{\pm }$ as well as on the surface *S*, the partial mass balances are

(16a) $$\partial_{t}\rho_{\alpha }+\mathrm{div}\left(\rho_{\alpha }\upsilon +J_{\alpha }\right)=r_{\alpha },\;\mathrm{in}\;\Omega^{\pm }\;\;\mathrm{for}\;\alpha \in I^{\pm }\;,$$

(16b) $$\partial_{t,\nu }\underset{s}{\rho }\;{\;}_{\alpha }+{\left(\underset{s}{\rho }\;{\;}_{\alpha }\underset{s}{\upsilon }\;{\;}_{\tau }^{\Delta }+\underset{s}{J}\;{\;}_{\alpha ,\tau }^{\Delta }\right)}_{∥\Delta }-2k_{M}\underset{s}{\upsilon }\;{\;}_{\nu }\;\underset{s}{\rho }\;{\;}_{\alpha }=\underset{s}{r}\;{\;}_{\alpha }-\left[\;\left[\rho_{\alpha }\left(\upsilon_{\nu }-\underset{s}{\upsilon }\;{\;}_{\nu }\right)+J_{\alpha }\cdot \nu \right]\;\right]\;\mathrm{on}\;S\;\;\mathrm{for}\;\alpha \in I_{S}\;.$$

Herein, $r_{\alpha }$ and $\underset{s}{r}\;{\;}_{\alpha }$ denote the mass production rate of constituent $A_{\alpha }$. They are defined by the reaction rates,

(17) $$\begin{array}{cccc} r_{\alpha } & =\sum_{k=1}^{M}m_{\alpha }\gamma_{\alpha }^{k}R^{k}\;, & \underset{s}{r}\;{\;}_{\alpha } & =\sum_{k=1}^{M_{S}}m_{\alpha }\underset{s}{\gamma }\;{\;}_{\alpha }^{k}\underset{s}{R}\;{\;}^{k}\;. \end{array}$$

The bulk and surface diffusion flux $J_{\alpha }$ and $\underset{s}{J}\;{\;}_{\alpha }$ with respect to the barycentric velocities are defined as

(18a) $$\begin{array}{ccc} J_{\alpha } & =\rho_{\alpha }\left(\upsilon_{\alpha }-\upsilon \right)\;, & \;\;\mathrm{implying}\;\;\sum_{\alpha \in I^{\pm }}J_{\alpha }=0\;, \end{array}$$

(18b) $$\begin{array}{ccc} \underset{s}{J}\;{\;}_{\alpha } & =\underset{s}{\rho }\;{\;}_{\alpha }\left(\underset{s}{\upsilon }\;{\;}_{\alpha }-\underset{s}{\upsilon }\;\;\right)\;, & \;\;\mathrm{implying}\;\;\sum_{\alpha \in I_{S}}\underset{s}{J}\;{\;}_{\alpha }=0\;. \end{array}$$

The partial mass balances can be combined to derive conservation laws for the total mass density of the mixture in the bulk and on the surface. In later applications, often it turns out that it is advantageous to replace partial mass balances by these conservation laws. From balances (16) together with the constraints (18) and (8), we obtain the conservation of total mass

(19a) $$\partial_{t}\rho +\mathrm{div}\left(\rho \upsilon \right)=0\;\mathrm{in}\;\Omega^{\pm }\;,$$

(19b) $$\partial_{t,\nu }\underset{s}{\rho }\;\;+{\left(\underset{s}{\rho }\;\;\underset{s}{\upsilon }\;{\;}_{\tau }^{\Delta }\right)}_{∥\Delta }-2k_{M}\underset{s}{\upsilon }\;{\;}_{\nu }\;\underset{s}{\rho }\;\;=-[\;\left[\rho \left(\upsilon_{\nu }-\underset{s}{\upsilon }\;{\;}_{\nu }\right)\right]\;]\;\mathrm{on}\;S\;.$$

Multiplication of the partial mass balances (16) by $z_{\alpha }e_{0}/m_{\alpha }$ and summation over all species together with conditions (8) implies conservation laws for free charge,

(20a) $$\begin{array}{ccc} \partial_{t}n^{F}+\mathrm{div}\left(n^{F}\upsilon +J^{F}\right) & =0 & \mathrm{in}\;\Omega^{\pm }\;, \end{array}$$

(20b) $$\begin{array}{ccc} \partial_{t,\nu }\underset{s}{n}\;{\;}^{F}+{\left(\underset{s}{n}\;{\;}^{F}\underset{s}{\upsilon }\;{\;}_{\tau }^{\Delta }+\underset{s}{J}\;{\;}_{\tau }^{F,\Delta }\right)}_{∥\Delta }-2k_{M}\underset{s}{\upsilon }\;{\;}_{\nu }\;\underset{s}{n}\;{\;}^{F}\; & =-[\;\left[n^{F}\left(\upsilon_{\nu }-\underset{s}{\upsilon }\;{\;}_{\nu }\right)+J_{\nu }^{F}\right]\;] & \mathrm{on}\;S, \end{array}$$

with the (non-convective) free electric current densities

(21) $$\begin{array}{cccc} J^{F} & =\sum_{\alpha \in I}\frac{z_{\alpha }e_{0}}{m_{\alpha }}J_{\alpha }\;, & \underset{s}{J}\;{\;}^{F} & =\sum_{\alpha \in I_{S}}\frac{z_{\alpha }e_{0}}{m_{\alpha }}\underset{s}{J}\;{\;}_{\alpha }\;. \end{array}$$

#### Balance of Momentum

The total momentum density ρυ of the mixture in bulk changes due to the momentum flux, which is given by the *(Cauchy) stress tensor* σ, as well as by the *gravitational force density* ρf and the *Lorentz force* k. Accordingly, the surface momentum density $\underset{s}{\rho }\;\;\underset{s}{\upsilon }\;\;$ changes due to the momentum flux $\underset{s}{\sigma }\;\;$, the *gravitational force density* $\underset{s}{\rho }\;\;\underset{s}{f}\;\;$ and the *Lorentz force* $\underset{s}{k}\;\;$. The balances of momentum for bulk and surface read

(22a) $$\begin{array}{ccc} \partial_{t}\rho \upsilon +\mathrm{div}\left(\rho \upsilon ⊗\upsilon -\sigma \right) & =\rho f+k & \mathrm{in}\;\Omega^{\pm }\;, \end{array}$$

(22b) $$\begin{array}{ccc} \partial_{t,\nu }\left(\underset{s}{\rho }\;\;\underset{s}{\upsilon }\;{\;}^{i}\right)+{\left(\underset{s}{\rho }\;\;\underset{s}{\upsilon }\;{\;}^{i}\underset{s}{\upsilon }\;{\;}_{\tau }^{\Delta }-\underset{s}{\sigma }\;{\;}^{i\Delta }\right)}_{∥\Delta }-2k_{M}\underset{s}{\upsilon }\;{\;}_{\nu }\;\underset{s}{\rho }\;\;\underset{s}{\upsilon }\;{\;}^{i} & =\underset{s}{\rho }\;\;\underset{s}{f}\;{\;}^{i}+\underset{s}{k}\;{\;}^{i}-\left[\;\left[\rho \upsilon^{i}\left(\upsilon_{\nu }-\underset{s}{\upsilon }\;{\;}_{\nu }\right)-\sigma^{ij}\nu_{j}\right]\;\right] & \mathrm{on}\;S\;. \end{array}$$

The momentum flux $\underset{s}{\sigma }\;\;$ is decomposed into its normal and tangential components

(23) $$\underset{s}{\sigma }\;{\;}^{i\Delta }=S^{\Gamma \Delta }\tau_{\Gamma }^{i}+S^{\Delta }\nu^{i}\;.$$

The tensor with the components $S^{\Gamma \Delta }$ is denoted as *surface stress tensor* and the vector with the components $S^{\Delta }$ is the *normal stress vector*. We neglect internal spin. This implies symmetry of the stress tensors and vanishing of the normal stress [21], i.e., for i,j=1,2,3 and Γ,Δ=1,2, we assume

(24) $$\sigma^{ij}=\sigma^{ji}\;,\;S^{\Gamma \Delta }=S^{\Delta \Gamma }\;\;\mathrm{and}\;\;S^{\Delta }=0\;.$$

#### Balance of Energy

The energy density of matter can be split into the internal energy density ρu and the kinetic energy density $\frac{1}{2}\rho {\left|\upsilon \right|}^{2}$. The splitting originates from the observation that the kinetic part of the energy can be eliminated by a suitable coordinate transformation whereas the internal energy cannot. This decomposition also implies a decomposition of the fluxes and the external sources into internal and kinetic contributions. The energy balance in the bulk and on the surface read

(25a) $$\partial_{t}(\rho u+\frac{1}{2}\rho {\left|\upsilon \right|}^{2})+\mathrm{div}\left((\rho u+\frac{1}{2}\rho |\upsilon {|}^{2})\upsilon +q-\upsilon \sigma \right)=\pi +\rho f\cdot \upsilon \;\mathrm{in}\;\Omega^{\pm }\;,$$

(25b) $$\begin{array}{cc} & \partial_{t,\nu }\left(\underset{s}{\rho }\;\;\underset{s}{u}\;\;+\frac{1}{2}\underset{s}{\rho }\;\;{\left|\underset{s}{\upsilon }\;\;\right|}^{2}\right)+{\left((\underset{s}{\rho }\;\;\underset{s}{u}\;\;+\frac{1}{2}\underset{s}{\rho }\;\;|\underset{s}{\upsilon }\;\;{|}^{2})\underset{s}{\upsilon }\;{\;}_{\tau }^{\Delta }+\underset{s}{q}\;{\;}^{\Delta }-\underset{s}{\sigma }\;{\;}^{i\Delta }\underset{s}{\upsilon }\;{\;}_{i}\right)}_{∥\Delta }-2k_{M}\underset{s}{\upsilon }\;{\;}_{\nu }\;\left(\underset{s}{\rho }\;\;\underset{s}{u}\;\;+\frac{1}{2}\underset{s}{\rho }\;\;{\left|\underset{s}{\upsilon }\;\;\right|}^{2}\right)\\ = & \underset{s}{\pi }\;\;+\underset{s}{\rho }\;\;\underset{s}{f}\;\;\cdot \underset{s}{\upsilon }\;\;-[\;[(\rho u+\frac{1}{2}{\rho |\upsilon |}^{2})\left(\upsilon_{\nu }-\underset{s}{\upsilon }\;{\;}_{\nu }\right)+\left(q-\upsilon \sigma \right)\cdot \nu ]\;]\;\;\mathrm{on}\;S. \end{array}$$

Here, q, $\underset{s}{q}\;\;$ denote the *heat fluxes* and π, $\underset{s}{\pi }\;\;$ are the *Joule heat* for bulk and surface, respectively.

### 3.2. Electromagnetic Fields

Maxwell’s equations for the electromagnetic field are based on two conservation laws: conservation of electric charge and conservation of magnetic flux, cf. [21,25].

#### Conservation of Electric Charge

The conservation equations of the total electric charge densities $n^{e}$ and $\underset{s}{n}\;{\;}^{e}$ in the bulk and on the surface read

(26a) $$\partial_{t}n^{e}+\mathrm{div}\left(n^{e}\upsilon +J^{e}\right)=0\;\mathrm{in}\;\Omega^{\pm }\;,$$

(26b) $$\partial_{t,\nu }\underset{s}{n}\;{\;}^{e}+{\left(\underset{s}{n}\;{\;}^{e}\underset{s}{\upsilon }\;{\;}_{\tau }^{\Delta }+\underset{s}{J}\;{\;}_{\tau }^{e,\Delta }\right)}_{∥\Delta }-2k_{M}\underset{s}{\upsilon }\;{\;}_{\nu }\;\underset{s}{n}\;{\;}^{e}\;=-[\;\left[n^{e}\left(\upsilon_{\nu }-\underset{s}{\upsilon }\;{\;}_{\nu }\right)+J_{\nu }^{e}\right]\;]\;\mathrm{on}\;S\;.$$

Here, the total electric current flux densities are split into a convective and non-convective part, i.e., $j^{e}=n^{e}\upsilon +J^{e}$ and $\underset{s}{j}\;{\;}^{e}=\underset{s}{n}\;{\;}^{e}\underset{s}{\upsilon }\;\;+\underset{s}{J}\;{\;}^{e}$. Then, we introduce the *charge potential* D and the *current potential* H as formal solutions of these charge balances,

(27a) $$n^{e}=\mathrm{div}D\;,\;J^{e}=-\partial_{t}D-\upsilon \mathrm{div}D+\mathrm{curl}H\;\mathrm{in}\;\Omega^{\pm }\;,$$

(27b) $$\underset{s}{n}\;{\;}^{e}=[\;[D\cdot \nu ]\;]\;,\;\underset{s}{J}\;{\;}^{e}=\nu \times [\;\left[H-\underset{s}{\upsilon }\;\;\times D\right]\;]\;\mathrm{on}\;S\;.$$

When considering the conservation of the free charge $n^{F}$, the same argumentation leads to a formulation of Maxwell’s equations in terms of the *electric displacement* field $D=\varepsilon_{0}E+P$ and the *magnetic field density* $H=\frac{1}{\mu_{0}}B-M$, instead of D and H. Here, P and M are the polarization and magnetization, respectively, that we introduce later in Section 3.3. In contrast to, e.g., [26], our derivation of Equation (27b) in Appendix A is not based on some averaging technique for the bulk equations.

#### Conservation of Magnetic Flux

The volume- and surface equations for the *electric field* E and the *magnetic flux density* B are derived from the conservation law of magnetic flux,

(28a) $$\mathrm{div}B=0\;,\;\partial_{t}B+\mathrm{curl}E=0\;\mathrm{in}\;\Omega^{\pm }\;,$$

(28b) $$[\;[B\cdot \nu ]\;]=0\;,\;\nu \times [\;\left[E+\underset{s}{\upsilon }\;\;\times B\right]\;]=0\;\mathrm{on}\;S\;.$$

#### Maxwell–Lorentz Aether Relations

The equation system (27) and (28) together constitute the system of Maxwell’s equations and boundary conditions. The system is underdetermined such that additional relations between (B,E) and (D,H) are needed. Since all material dependence is incorporated into the total charge density $n^{e},\underset{s}{n}\;{\;}^{e}$, we can postulate universal valid Maxwell–Lorentz aether relations [21,25,27],

(29) $$D=\varepsilon_{0}E\;\mathrm{and}\;H=\frac{1}{\mu_{0}}B\;.$$

Here, $\varepsilon_{0}$ is the *dielectric constant* and $\mu_{0}$ is the *magnetic constant*. They are related to the speed of light by $\varepsilon_{0}\mu_{0}=c_{0}^{-2}\;.$

### 3.3. Coupling of Equations for Matter and Electrodynamics

The coupling of the equations of matter in Section 3.1 and Maxwell’s equations according to Section 3.2 is done in the following two steps. First, we identify the balances for *polarization* and *magnetization*. Second, by requiring conservation of total energy and total momentum, we identify the Lorentz force and Joule heat as functions of the electromagnetic fields.

#### 3.3.1. Polarization and Magnetization

The conservation laws (26) for the total charge and (20) for the free charge imply also conservation of the polarization charge $n^{P}=n^{e}-n^{F}$, $\underset{s}{n}\;{\;}^{P}=\underset{s}{n}\;{\;}^{e}-\underset{s}{n}\;{\;}^{F}$, i.e.,

(30a) $$\partial_{t}n^{P}+\mathrm{div}\left(n^{P}\upsilon +J^{P}\right)=0\;\mathrm{in}\;\Omega^{\pm }\;,$$

(30b) $$\partial_{t,\nu }\underset{s}{n}\;{\;}^{P}+{\left(\underset{s}{n}\;{\;}^{P}\underset{s}{\upsilon }\;{\;}_{\tau }^{\Delta }+\underset{s}{J}\;{\;}_{\tau }^{P,\Delta }\right)}_{∥\Delta }-2k_{M}\underset{s}{\upsilon }\;{\;}_{\nu }\;\underset{s}{n}\;{\;}^{P}\;=-\left[\;\left[n^{P}\left(\upsilon_{\nu }-\underset{s}{\upsilon }\;{\;}_{\nu }\right)\right]\;\right]-\left[\;\left[J_{\nu }^{P}\right]\;\right]\;\mathrm{on}\;S\;,$$

with the *polarization current densities* according to

(31) $$J^{e}=J^{F}+J^{P}\;,\;\underset{s}{J}\;{\;}^{e}=\underset{s}{J}\;{\;}^{F}+\underset{s}{J}\;{\;}^{P}\;.$$

Motivated by the introduction of D and H as formal solution of the balance equations (26), we use an analogous approach to introduce *polarization* P and *Lorentz magnetization* M by formal solution of the balance equations (30) according to Appendix A. We get

(32a) $$n^{P}=-\mathrm{div}(P)\;,\;J^{P}=\partial_{t}P+\upsilon \mathrm{div}P+\mathrm{curl}M\;\mathrm{in}\;\Omega^{\pm }\;,$$

(32b) $$\underset{s}{n}\;{\;}^{P}=-[\;[P]\;]\cdot \nu \;,\;\underset{s}{J}\;{\;}^{P}=\nu \times [\;\left[M+\underset{s}{\upsilon }\;\;\times P\right]\;]\;\mathrm{on}\;S\;.$$

This introduction of the fields P and M differs from the classical textbook literature, where often microscopic models are used, e.g., P is introduced by considering microscopic electric dipoles and M is derived from microscopic circular currents. Then, the polarization charge $n^{P}$ and polarization currents $J^{P}$ are formally introduced to couple P and M to the Maxwell’s equations, cf. [21,27,28,29,30,31]. Our approach leads to the same relations between ($n^{P}$, $J^{P}$) and (P, M), but it has the advantages that it is independent from any microscopic model and, most notably, its ability to transfer the concept of polarization and magnetization to the surface. The relation of the fields (P,M) to (E,B) is left to a subsequent material modeling.

For the fields P and M in the bulk, there are no according counterparts on the surface in our approach. An extension of the theory seems to be possible. For instance in [32], surface polarization and magnetization are derived from the bulk Maxwell’s equation by integration over a singularity and thus for each bulk quantity there exists a corresponding surface quantity. While in our approach P and M are introduced as a particular formal solution of the balance equation for polarization charge, there may be a further formal solution of the surface balance equation that allows the introduction of surface polarization vector and surface magnetization. Although we did not introduce a surface polarization vector or surface magnetization, there exists surface polarization charge $\underset{s}{n}\;{\;}^{P}$ and surface flux densities $\underset{s}{J}\;{\;}^{P}$ due to polarization and magnetization, cf. Equation (32b).

Due to its importance in electrodynamics and for the upcoming constitutive modelling, we introduce the *electromotive intensity* and the *magnetization*

(33) E=E+υ×B,        M=M+υ×P.

#### 3.3.2. Lorentz Force and Joule Heat

From Maxwell’s equations (27) and (28), two more balance equations can be derived: the balance of the electromagnetic momentum and the balance of the energy of the electromagnetic field.

##### Balance of Electromagnetic Energy

The electromagnetic energy density in the bulk is defined as $\frac{1}{2}\left(E\cdot D+B\cdot H\right)$. From Maxwell’s equations, we get the balance equations, [21,26]:

(34a) $$\frac{1}{2}\partial_{t}\left(E\cdot D+B\cdot H\right)+\mathrm{div}\left(E\times H\right)=-(n^{e}\upsilon +J^{e})\cdot E\;\mathrm{in}\;\Omega^{\pm }\;,$$

(34b) $$-\left[\;\left[\frac{1}{2}\left(E\cdot D+B\cdot H\right)\underset{s}{\upsilon }\;{\;}_{\nu }\right]\;\right]+\left[\;\left[\left(E\times H\right)\cdot \nu \right]\;\right]=-\left(\underset{s}{n}\;{\;}^{e}\underset{s}{\upsilon }\;\;+\underset{s}{J}\;{\;}^{e}\right)\cdot \bar{E}\;\mathrm{on}\;S\;.$$

Here, E×H is the *electromagnetic energy flux density* (Poynting vector) and $(n^{e}\upsilon +J^{e})\cdot E$ is the energy gained by the matter due to the electromagnetic field. On the surface, there is no additional electromagnetic surface energy density/flux implied by Maxwell’s equations. Thus, the influxes from the bulk are solely balanced by the surface production $\left(\underset{s}{n}\;{\;}^{e}\underset{s}{\upsilon }\;\;+\underset{s}{J}\;{\;}^{e}\right)\cdot \bar{E}$.

##### Balance of Electromagnetic Momentum

The electromagnetic momentum density is defined as D×B. Maxwell’s equations imply in bulk and surface the balances for electromagnetic momentum [21,26]:

(35a) $$\partial_{t}\left(D\times B\right)-\mathrm{div}\left(H⊗B+E⊗D-\frac{1}{2}\left(H\cdot B+D\cdot E\right)I\right)=-n^{e}E-\left(n^{e}\upsilon +J^{e}\right)\times B\;\mathrm{in}\;\Omega^{\pm }\;,$$

(35b) $$-\left[\;\left[\left(D\times B\right)\underset{s}{\upsilon }\;{\;}_{\nu }\right]\;\right]+\left[\;\left[HB_{\nu }+ED_{\nu }+\frac{1}{2}\left(H\cdot B+E\cdot D\right)\nu \right]\;\right]=-\underset{s}{n}\;{\;}^{e}\bar{E}-\left(\underset{s}{n}\;{\;}^{e}\underset{s}{\upsilon }\;\;+\underset{s}{J}\;{\;}^{e}\right)\times \bar{B}\;\mathrm{on}\;S\;.$$

The electromagnetic momentum flux density $\left(H⊗B+E⊗D\right)-\frac{1}{2}\left(H\cdot B+E\cdot D\right)I$ is called the *Maxwell stress tensor*. On the right-hand sides of (35), the productions $n^{e}E+\left(n^{e}\upsilon +J^{e}\right)\times B$ and $\underset{s}{n}\;{\;}^{e}\bar{E}+\left(\underset{s}{n}\;{\;}^{e}\underset{s}{\upsilon }\;\;+\underset{s}{J}\;{\;}^{e}\right)\times \bar{B}$ define the force densities due to the electromagnetic field. Like before in (34), Maxwell’s equations (27) and (28) do not imply surface momentum density/flux, such that the bulk influxes are balanced by the surface production.

##### Identification of Lorentz Force and Joule Heat

We postulate that, in the absence of gravitation, i.e., for $f=\underset{s}{f}\;\;=0$, the *total momentum* ρυ+(D×B) and the *total energy* $\rho (u+\frac{1}{2}{\left|\upsilon \right|}^{2})+\frac{1}{2}\left(E\cdot D+B\cdot H\right)$ of matter and electromagnetic field are conserved quantities, cf. [21,25,26]. Then, the production in the balance equations of matter have to be canceled by the electromagnetic productions. This implies for the Lorentz force $k,\underset{s}{k}\;\;$ and the Joule heat $\pi ,\underset{s}{\pi }\;\;$

(36a) $$\pi =(n^{e}\upsilon +J^{e})\cdot E\;,\;k=n^{e}E+\left(n^{e}\upsilon +J^{e}\right)\times B\;\mathrm{in}\;\Omega^{\pm }\;,$$

(36b) $$\underset{s}{\pi }\;\;=\left(\underset{s}{n}\;{\;}^{e}\underset{s}{\upsilon }\;\;+\underset{s}{J}\;{\;}^{e}\right)\cdot \bar{E}\;,\;\underset{s}{k}\;\;=\underset{s}{n}\;{\;}^{e}\bar{E}+\left(\underset{s}{n}\;{\;}^{e}\underset{s}{\upsilon }\;\;+\underset{s}{J}\;{\;}^{e}\right)\times \bar{B}\;\mathrm{on}\;S\;.$$

While the definition of mixture quantities like the total energy and total momentum in the authors point of view should not be modified, terms in the balance equations can be moved from the fluxes to the productions and vice versa. Discussions in literature on the Lorentz force and Joule heat, cf. e.g., [31], mostly originate from this ambiguity.

#### 3.3.3. Balances of Total Momentum and Internal Energy

As a consequence of (36), we get the total momentum balances

(37a) $$\partial_{t}\left(\rho \upsilon +D\times B\right)+\mathrm{div}\left(\rho \upsilon ⊗\upsilon -\Sigma \right)=\rho f\;\mathrm{in}\;\Omega^{\pm }\;,$$

(37b) $$\partial_{t,\nu }\left(\underset{s}{\rho }\;\;\underset{s}{\upsilon }\;{\;}^{i}\right)+{\left(\underset{s}{\rho }\;\;\underset{s}{\upsilon }\;{\;}^{i}\underset{s}{\upsilon }\;{\;}_{\tau }^{\Delta }-\underset{s}{\sigma }\;{\;}^{i\Delta }\right)}_{∥\Delta }-2k_{M}\underset{s}{\upsilon }\;{\;}_{\nu }\;\underset{s}{\rho }\;\;\underset{s}{\upsilon }\;{\;}^{i}=\underset{s}{\rho }\;\;\underset{s}{f}\;{\;}^{i}-\left[\;\left[\rho \upsilon^{i}\left(\upsilon_{\nu }-\underset{s}{\upsilon }\;{\;}_{\nu }\right)-\Sigma^{ij}\nu_{j}\right]\;\right]\;\mathrm{on}\;S\;,$$

where the *total stress tensor* consisting of Cauchy and Maxwell stress is given by

(38) $$\Sigma =\sigma +H⊗B+E⊗D-\frac{1}{2}\left(H\cdot B+D\cdot E\right)1\;,$$

where 1 denotes the identity matrix. In the absence of the gravitational force and assuming stationarity and vanishing velocity, the total momentum balances reduces to

(39) $$\mathrm{Equilibrium}:\;\;\mathrm{div}\Sigma =0\;,\;[\;[\Sigma \nu ]\;]=-\underset{s}{\sigma }\;{\;}_{∥\Delta }^{i\Delta }\;.$$

In particular, if the surface stress vanishes, then the total stress is continuous at the surface. Therefore, one can only measure the total stress, but not separately the Cauchy stress or the Maxwell stress. This fact can be nicely studied in an experiment with two capacitor plates dipped into a liquid, cf. ([29], Section 3.6).

For the later exploitation of the entropy principle, we derive an appropriate form of the balance of internal energy. The internal energy balance is derived in three steps: first, the kinetic energy is eliminated in the energy balance (25) by means of the momentum balance (22). Second, the electromagnetic contributions are split into a free and polarization part. Third, the polarization current is replaced by using the identities for the polarization vector and Lorentz magnetization (32b):

(40a) $$\begin{array}{cc} \frac{\partial (\rho u+M\cdot B)}{\partial t} & +\mathrm{div}\left((\rho u+M\cdot B)\upsilon +q+(E\times M)\right)\\ =\; & \left(\sigma +M⊗B-E⊗P+(E\cdot P)1\right):\nabla \upsilon \\ & +E\cdot J^{F}+\left(\frac{\partial P}{\partial t}+\left(\upsilon \cdot \nabla \right)P\right)\cdot E+\left(\frac{\partial M}{\partial t}+\left(\upsilon \cdot \nabla \right)M\right)\cdot B\;\mathrm{in}\;\Omega^{\pm }\;, \end{array}$$

(40b) $$\begin{array}{cc} \partial_{t,\nu }\left(\underset{s}{\rho }\;\;\underset{s}{u}\;\;\right) & +{\left(\underset{s}{\rho }\;\;\underset{s}{u}\;\;\;\underset{s}{\upsilon }\;{\;}_{\tau }^{\Delta }+\underset{s}{q}\;{\;}^{\Delta }\right)}_{∥\Delta }-2k_{M}\underset{s}{\upsilon }\;{\;}_{\nu }\;\underset{s}{\rho }\;\;\underset{s}{u}\;\;\\ \;=\; & \underset{s}{\sigma }\;{\;}^{i\Delta }\underset{s}{\upsilon }\;{\;}_{∥\Delta }^{i}+\left(\bar{E}+\underset{s}{\upsilon }\;\;\times \bar{B}\right)\cdot \underset{s}{J}\;{\;}^{F}\\ & -\left[\;\;\left[\left(u+\frac{1}{2}{\left|\underset{s}{\upsilon }\;\;-\upsilon \right|}^{2}\right)\rho \left(\upsilon_{\nu }-\underset{s}{\upsilon }\;{\;}_{\nu }\right)-{\left(\upsilon -\underset{s}{\upsilon }\;\;\right)}^{T}\sigma \nu +\left(q+E\times M\right)\cdot \nu \right]\;\;\right]\\ & +\left[\;\;\left[\left(P\left(\upsilon_{\nu }-\underset{s}{\upsilon }\;{\;}_{\nu }\right)-P_{\nu }\left(\upsilon -\underset{s}{\upsilon }\;\;\right)\right)\cdot E\right]\;\;\right]+\left(P\times B\right)\cdot \left(\upsilon -\underset{s}{\upsilon }\;\;\right)\left(\upsilon_{\nu }-\underset{s}{\upsilon }\;{\;}_{\nu }\right)\\ & -\left[\;\;\left[\left(B\left(\upsilon_{\nu }-\underset{s}{\upsilon }\;{\;}_{\nu }\right)-B_{\nu }\left(\upsilon -\underset{s}{\upsilon }\;\;\right)\right)\cdot M\right]\;\;\right]\mathrm{on}\;S\;. \end{array}$$

## 4. Symmetry Principles for Observer Transformations

In this section, we study changes of space and time coordinates of the observer frame of reference, shortly called observer transformations. Their application to the fields of thermodynamics and electrodynamics show diverse transformation properties leading to symmetry principles that particularly restrict the generality of the admissible constitutive functions.

In the previous section, the equations of balance for matter, the conservation law of charge, the magnetic flux conservation and the Maxwell–Lorentz aether relations were formulated with respect to an *inertial frame* of reference. Time and spatial coordinates refer to an inertial frame if two conditions are met: (1) the mass center of a material body that is not subjected to external forces moves with constant (barycentric) velocity along a straight line, and (2) the Maxwell–Lorentz aether relations hold.

### Symmetry Principles

The most fundamental symmetry principle is the *principle of relativity*. It restricts balance equations as well as constitutive equations by stating invariance with respect to arbitrary observer transformations. However, this principle can only be maintained if time and space and all involved equations are properly combined to four-dimensional objects.

We will not touch the four-dimensional case further and restrict ourselves to a less general symmetry principle suitable for the 1+3-dimensional case. In the following, we introduce and exploit the *Galilean symmetry principle* stating invariance of the involved equations with respect to Galilean transformations defined below in Equation (41). It is well known that the balance equations of matter (16)–(25) are invariant with respect to Galilean transformations. On the other hand, the Maxwell equations are invariant with respect to arbitrary transformations, including Galilean transformations as well. However, the 1+3-dimensional Maxwell–Lorentz aether relations are only invariant with respect to Lorentz transformations. Nevertheless, in the limit of vanishing barycentric velocity, i.e., v/c→0, the Galilean transformation is a good approximation of the Lorentz transformation. However, even in this case, one should be aware that by applying the Galilean symmetry principle, important classical effects are ignored. Two examples are: (1) Galilean invariance is intimately linked to the conservation of mass in a chemical reaction, thus we cannot describe the case of atomic fusion in this setting. (2) The unipolar generator cannot be explained with models restricted by the Galilean symmetry principle. A relativistic explanation is needed. For the electrochemical applications that we have in mind, these inconsistencies between the Maxwell–Lorentz aether relations and the Galilean symmetry principle are not relevant and are not further discussed here.

Finally, we mention the *Euclidean transformation*, which is another classical transformation that generalizes the Galilean transformation. For these transformations, often invariance is proposed with respect to constitutive functions only, and the corresponding symmetry principle is called *material frame indifference*. In this paper, we do not consider that concept any further.

In summary, we may say that the general essentials of symmetry principles for observer transformation consist of three steps: (i) We choose a class of space-time transformation. (ii) We investigate the transformation properties of the involved quantities under that transformation. (iii) We propose a corresponding symmetry principle stating invariance of certain equations, for example invariance of the constitutive equations.

### Galilean Symmetry Principle

For a Galilean transformation, the transformed coordinates are given by

(41) $$\bar{t}=t,\;\bar{x}=Ox-Wt\;\mathrm{with}\;O^{T}O=I\;,$$

where the orthogonal matrix O and the velocity W are time independent.

We now introduce the *principle of Galilean symmetry* that states: The equations of balance for matter, Maxwell’s equations and the constitutive functions must be invariant with respect to the Galilean transformation (41).

The fields $T_{i_{1},i_{2},...,i_{N}}$ are identified as the components of a Galilean tenor (of rank *N*) if they transform under Galilean transformations according to

(42) $${\bar{T}}^{i_{1},i_{2},\ldots ,i_{N}}=\det{\left(O\right)}^{p}O_{i_{1}j_{1}}O_{i_{2}j_{2}}\ldots O_{i_{N}j_{N}}T^{j_{1},j_{2},\ldots ,j_{N}}.$$

For p=1, T is called an *axial Galilean tensor* while p=0 indicates an *absolute Galilean tensor*. The special cases N=0,1 refer to Galilean scalars and Galilean vectors, respectively.

### Classification of the Involved Fields

For the velocity, the deformation and the tangential vectors of the surface, it is possible to derive the transformation properties with respect to Galilean transformations as

(43a) $$\upsilon ,\;\underset{s}{\upsilon }\;\;,\;F,\;\tau_{\Delta }\;\mathrm{absolute}\;\mathrm{vectors},$$

(43b) $$\nabla v+{\left(\nabla v\right)}^{T}\;\mathrm{absolute}\;\mathrm{tensor}.$$

Next, we classify the involved fields in the balance equations for matter, such that these remain invariant under Galilean transformations, i.e.,

(44a) $$\rho_{\alpha },\;\underset{s}{\rho }\;{\;}_{\alpha },\;\rho u,\;\underset{s}{\rho }\;\;\underset{s}{u}\;\;,\;\;R^{k},\;\underset{s}{R}\;{\;}^{k}\;\mathrm{absolute}\;\mathrm{scalars},$$

(44b) $$J_{\alpha },\;\underset{s}{J}\;{\;}_{\alpha },\;q,\;\underset{s}{q}\;\;\;\mathrm{absolute}\;\mathrm{vectors},$$

(44c) σ,Σabsolutetensor.

In the four-dimensional formulation, Maxwell’s equations are invariant under arbitrary observer transformation. From these, the transformation properties of the electromagnetic fields in the 1+3-dimensional setting can be derived. The electromagnetic fields E,H and M are not vectors under a Galilean transformation, but specific combinations of these are, i.e.,

(45a) $$n^{e},\;\underset{s}{n}\;{\;}^{e}\;\mathrm{absolute}\;\mathrm{scalars},$$

(45b) $$J^{e},\;\underset{s}{J}\;{\;}^{e},\;\left(E+v\times B\right),\;\left(E+\underset{s}{v}\;\;\times B\right),\;D,\;P\;\mathrm{absolute}\;\mathrm{vectors},$$

(45c) $$B,\left(H-v\times D\right),\;\left(H-\underset{s}{v}\;\;\times D\right),\;\left(M+v\times P\right),\;\left(M+\underset{s}{v}\;\;\times P\right)\;\mathrm{axial}\;\mathrm{vectors}.$$

## 5. The Entropy Principle

The system of balance equations from Section 3 has to be closed by constitutive equations in the bulk and on the surface. The—thus far undetermined—constitutive quantities are the partial mass fluxes $J_{\alpha },\underset{s}{J_{\alpha }}\;\;$, reaction rates $R^{k},\underset{s}{R}\;{\;}^{k}$, heat fluxes $q,\underset{s}{q}\;\;$ stress tensors σ,S, polarization P and magnetization M. The constitutive equations are not uniquely determined, but they are restricted by the second law of thermodynamics, which is here expressed in terms of the entropy principle, and the principle of Galilean symmetry. We here follow the strictly axiomatic approach of [24] where also slightly different forms in the literature are reviewed, cf. e.g., [20,21,33,34]. The formulation of the entropy principle is largely analogous in the bulk and on the surface, and therefore done simultaneously.

### 5.1. Formulation of the Entropy Principle

The entropy principle consists of five axioms, of which the first part consisting of axioms I–III is universal, i.e., independent from the considered material. The second part with the axioms IV–V is material dependent, although in a very general and abstract way, because an appropriate set of independent variables has to be chosen for the considered material. Here, we consider elastic, viscous, magnetizable, polarizable, heat conducting and reactive mixtures.

#### The Entropy Principle

I. The entropy densities $\rho \eta ,\underset{s}{\rho }\;\;\underset{s}{\eta }\;\;$, entropy fluxes $\phi ,\underset{s}{\phi }\;\;$ and entropy productions $\xi ,\underset{s}{\xi }\;\;$ satisfy the balance equations:
  (46a) $$\partial_{t}\rho \eta +\mathrm{div}\left(\rho \eta \upsilon +\phi \right)=\xi \;\mathrm{in}\;\Omega^{\pm }\;,$$
  (46b) $$\partial_{t,\nu }\left(\underset{s}{\rho }\;\;\underset{s}{\eta }\;\;\right)+\left(\underset{s}{\rho }\;\;\underset{s}{\eta }\;\;\underset{s}{\upsilon }\;{\;}_{t}^{\Delta }au+\underset{s}{\phi }\;{\;}_{\tau }^{\Delta }\right){}_{∥\Delta }-2k_{M}\underset{s}{\upsilon }\;{\;}_{\nu }\underset{s}{\rho }\;\;\underset{s}{\eta }\;\;=\underset{s}{\xi }\;\;-\left[\;\left[\rho \eta \left(\upsilon_{\nu }-\underset{s}{\upsilon }\;{\;}_{\nu }\right)+\phi_{\nu }\right]\;\right]\;\mathrm{on}\;S\;.$$
II. $\rho \eta ,\underset{s}{\rho }\;\;\underset{s}{\eta }\;\;$ and $\xi ,\underset{s}{\xi }\;\;$ are absolute scalars and $\phi ,\underset{s}{\phi }\;\;$ are absolute vectors with respect to Galilean transformations.
III. The entropy productions $\xi ,\underset{s}{\xi }\;\;$ satisfy
  (i) the entropy productions $\xi ,\underset{s}{\xi }\;\;$ are *non-negative* for each solution of the balance equations and Maxwell’s equations.
  (ii) the entropy productions have representations as sums of binary products
    (47) $$0\overset{!}{\leq }\xi =\sum_{A}N_{A}P_{A}\;,\;0\overset{!}{\leq }\underset{s}{\xi }\;\;=\sum_{A}\underset{s}{N}\;{\;}_{A}\underset{s}{P}\;{\;}_{A}\;.$$
    The factors of each product are either scalars, vectors or tensors with respect to Galilean transformations. Each product $N_{A}P_{A}$ resp. $\underset{s}{N}\;{\;}_{A}\underset{s}{P}\;{\;}_{A}$ can be associated with exactly one dissipation mechanism.
  (iii) The entropy productions vanish in equilibrium.
IV. The entropy densities $\rho \eta ,\underset{s}{\rho }\;\;\underset{s}{\eta }\;\;$ have representations as concave functions $\rho \tilde{\eta }$, $\underset{s}{\rho }\;\;\underset{s}{\tilde{\eta }}\;\;$ of the independent variables. For elastic, viscous, magnetizable, polarizable, heat conducting and reactive mixtures, the independent variables are $\rho u+M\cdot B,{\left(\rho_{\alpha }\right)}_{\alpha \in I^{\pm }},F^{\mathrm{uni}},P,M$ in the bulk and $\underset{s}{\rho }\;\;\underset{s}{u}\;\;,{\left(\underset{s}{\rho }\;{\;}_{\alpha }\right)}_{\alpha \in I_{S}},\tau_{1}^{\mathrm{uni}},\tau_{2}^{\mathrm{uni}}$ on the surface
  (48a) $$\rho \eta =\rho \tilde{\eta }\left(\rho u+M\cdot B,{\left(\rho_{\alpha }\right)}_{\alpha \in I^{\pm }},F^{\mathrm{uni}},P,M\right)\;,$$
  (48b) $$\underset{s}{\rho }\;\;\underset{s}{\eta }\;\;=\underset{s}{\rho }\;\;\underset{s}{\tilde{\eta }}\;\;\left(\underset{s}{\rho }\;\;\underset{s}{u}\;\;,{\left(\underset{s}{\rho }\;{\;}_{\alpha }\right)}_{\alpha \in I_{S}},\tau_{1}^{\mathrm{uni}},\tau_{2}^{\mathrm{uni}}\right)\;.$$
V. The (absolute) temperature $T,\underset{s}{T}\;\;$ and the chemical potentials $\mu_{\alpha },\underset{s}{\mu }\;{\;}_{\alpha }$ are defined as
  (49a) $$\frac{1}{T}=\frac{\partial \rho \tilde{\eta }}{\partial (\rho u+M\cdot B)}\;,\;\frac{\mu_{\alpha }}{T}=-\frac{\partial \rho \tilde{\eta }}{\partial \rho_{\alpha }}\;,$$
  (49b) $$\frac{1}{\underset{s}{T}\;\;}=\frac{\partial \underset{s}{\rho }\;\;\underset{s}{\tilde{\eta }}\;\;}{\partial \underset{s}{\rho }\;\;\underset{s}{u}\;\;}\;,\;\frac{\underset{s}{\mu }\;{\;}_{\alpha }}{\underset{s}{T}\;\;}=-\frac{\partial \underset{s}{\rho }\;\;\underset{s}{\tilde{\eta }}\;\;}{\partial \underset{s}{\rho }\;{\;}_{\alpha }}\;.$$

#### Remarks

Axiom IV contains a specific set of variables. The entropy principle can be formulated with different sets of variables as well, and these different choices will in general lead to different constitutive equations. In particular, we assume that the bulk entropy density depends on the internal energy ρu+M·B instead of ρu. This internal energy allows us to derive an entropy production, which satisfies Axiom III in particular the representation (47). Different other choices for the internal energy that allow a respective formulation of the entropy principle can be found in [31]. In Section 6.4, we show that not every choice of constitutive functions that satisfies Axiom III automatically admits relaxation to the equilibrium.

Axiom IV requires that the set variables for the entropy densities are independent from each other. This is not the case for the deformation gradient F and the tangential vectors $\tau_{\Delta }$, for Δ=1,2, since the mass balances (19) are solved by

(50) $$\rho =\frac{\rho^{\mathrm{ref}}}{\det(F)}\;,\;\underset{s}{\rho }\;\;=\frac{\underset{s}{\rho }\;{\;}^{\mathrm{ref}}}{\sqrt{\det(g)}}\;,$$

where $\rho^{\mathrm{ref}},\underset{s}{\rho }\;{\;}^{\mathrm{ref}}$ are time-independent constants and the total mass flux from the bulk is assumed to vanish, i.e., $[\;\left[\rho \left(\upsilon -\underset{s}{\upsilon }\;\;\right)\right]\;]=0$. Therefore, the deformation gradients and tangential vectors cannot be used in Axiom IV and they are replaced by their unimodular counterparts.

There is a difference between the constitutive equations of the entropy density for bulk and surface because in our approach there are no counterparts of the fields P and M on the surface.

### 5.2. Exploitation of the Entropy Principle—General Approach

The general strategy for the design of constitutive relations can be outlined in a material independent way. Using only the structure (47) of the entropy production, we derive thermodynamic consistent constitutive equations from either linear or nonlinear relations between the factors $N_{A}$ and $P_{A}$, such that the entropy production is non-negative.

For the ease of presentation, we restrict ourselves to the case that the factors of each binary product are scalars. A generalization to vectors and tensors can easily be done.

#### Linear Relations

To account for cross effects, we consider a regular matrix *M* and set $Q=M^{-1}$. Then, the entropy production (47) is rewritten as

(51) $$0\overset{!}{\leq }\xi =\sum_{A}N_{A}P_{A}=\sum_{A,B,C}\left(N_{A}Q_{AB}\right)\left(M_{BC}P_{C}\right)\;.$$

To have $\sum_{A}N_{A}Q_{AB}$ proportional to $\sum_{C}M_{BC}P_{C}$, we choose positive phenomenological coefficients $L_{B}>0$ and set

(52) $$K=M^{T}\mathrm{diag}\left(L_{B}\right)M\;\;\mathrm{and}\;\;N_{A}=\sum_{C}K_{AC}P_{C}\;,$$

where the coefficient matrix *K* is symmetric and positive definite by construction. The coefficients of the matrix *M* and $L_{B}$ in general may be functions of the independent variables as long as they are absolute scalars with respect to Galilean transformations. We want to stress here that, in the generality of the above formulation, the entropy principle provides no information about this dependencies on the independent variables apart from the requirements that $L_{B}\geq 0$ and *M* regular. Instead, theories of different nature or, like e.g., in [35,36], specific assumptions on the considered system are needed to derive such functional dependencies.

#### Nonlinear Relations

Thermodynamically consistent constitutive equations based on linear relation (51) can also be derived by nonlinear closure relations. Since chemical reactions are often modeled by an Arrhenius type exponential dependency of the reaction rate on the driving force, we thus propose the nonlinear relation

(53) $$N_{A}=-\sum_{B}M_{BA}L_{B}\left(1-\exp\left(K_{B}\sum_{C}M_{BC}P_{C}\right)\right)\;,$$

where the phenomenological coefficients $L_{B}$ and $K_{B}$ are positive. For a system close to equilibrium, such that $0\leq |K_{B}\sum_{C}M_{BC}P_{C}|\ll 1$, the exponential function in (53) may be linearized and the nonlinear closure relations (53) then approach the linear ones in (52).

#### Galilean Symmetry Principle

The constitutive relations that result from the above closure relations automatically satisfy the Galilean symmetry principle, since the factors of the binary products are scalars, vectors or tensors with respected to Galilean transformations according Axiom III (ii) and for the phenomenological coefficients only absolute scalars according to Section 4 are chosen.

#### Remark on Cross Effects and Symmetry of the Phenomenological Coefficient Matrix

The well known Onsager–Casimir reciprocal relations postulate the symmetry of the matrix *K* that appears in closure relation (52). They are motivated by experimental observations of thermo-electrical effects by Thomson ([37], pp. 237–241) and have been proven for systems of ordinary differential equations describing homogeneous processes in a statistical mechanics by Onsager and Casimir [33,38,39,40].

By the construction based on the matrix *M*, the symmetry of *K* is automatically guaranteed. Moreover, the introduction of *M* in the entropy production (51) allows in a very simple way the realization of cross effects in the nonlinear case. However, one should note that, as long as the factors $N_{A}$ and $P_{A}$ have not been specified, it is also possible to construct different linear closure relations such that the coefficient matrix *K* is positive definite, but anti-symmetric(!), cf. [24]. There, the concept of parity is introduced to solve these problems by further characterization of the factors $N_{A}$ and $P_{A}$. If the quantities $N_{A}$ exclusively consist of objects with physical units containing odd powers of the time unit and all objects of $P_{A}$ have units with even powers of the second, then we have symmetry of *K* by our construction. Now, it is easy to observe that an exchange of some $N_{A}$ objects with $P_{A}$ objects then yields antisymmetry of *K*.

## 6. Constitutive Equations for Magnetizable, Polarizable, Viscous and Reactive Mixtures

### 6.1. Constitutive Relations for the Bulk

To derive the bulk constitutive equations for the mass fluxes $J_{\alpha }$, reaction rates $R^{k}$, heat flux q, stress tensor σ, polarization P and magnetization M, we first identify the entropy production. To do so, we insert the entropy density (48a) into the entropy balance (46a) and apply the chain rule of differentiation. Then, the occurring time derivatives are substituted by means of the balance equations, where in particular we apply the balance of the internal energy (40a). At this stage, it is still possible to rearrange the terms in several ways. Once the entropy flux is fixed, the entropy production is uniquely determined. In order to get the structure (47), we choose

(54) $$\phi \;=\;\frac{q+E\times M}{T}-\sum_{\alpha \in I^{\pm }}\frac{\mu_{\alpha }}{T}J_{\alpha }\;.$$

Other constitutive equations for the entropy flux different from (54) can be admissible as well. In general, such alternative choices will lead to different constitutive equations for the entropy production. For a discussion of alternative choices of the electromagnetic contributions to the entropy density, we refer to Section 6.4 and in [41] a non-trivial example of generalized entropy fluxes in the context of viscous Cahn–Hilliard equations is discussed.

With the choice of the entropy flux (54), the entropy production is given by a sum of six binary products. We can identify six dissipation mechanisms related to their specific entropy production: shear viscosity $\xi_{SV}$, volume viscosity $\xi_{VV}$, (bulk-)reactions $\xi_{R}$, thermodiffusion $\xi_{TD}$, polarization $\xi_{P}$ and magnetization $\xi_{M}$. The entropy production is then given by the constitutive equation

(55) $$\begin{array}{ll} \xi =\; & \underset{=\xi_{SV}}{\underset{︸}{\frac{1}{T}\left(T-\frac{1}{3}\mathrm{trace}\left(T\right)1\right):\left(D-\frac{1}{3}\mathrm{trace}\left(D\right)1\right)}}+\underset{\xi_{VV}}{\underset{︸}{\frac{1}{3}\frac{1}{T}\mathrm{trace}\left(T\right)\mathrm{trace}\left(D\right)}}\\ & +\underset{\xi_{R}}{\underset{︸}{\frac{1}{T}\sum_{k=1}^{M}\left(-\sum_{\alpha \in I^{\pm }}\gamma_{\alpha }^{k}m_{\alpha }\mu_{\alpha }\right)R^{k}}}\\ & +\underset{=\xi_{TD}}{\underset{︸}{\left(q+(E\times M)\right)\cdot \nabla \;\left(\;\frac{1}{T}\;\right)\;-\;\sum_{\alpha \in I^{\pm }\backslash \left\{A_{0\pm }\right\}}J_{\alpha }\cdot \left(\nabla \left(\frac{\mu_{\alpha }}{T}-\frac{\mu_{0}}{T}\right)-\frac{1}{T}\;\left(\frac{z_{\alpha }e_{0}}{m_{\alpha }}-\frac{z_{0}e_{0}}{m_{0}}\right)E\right)}}\\ & +\underset{\xi_{P}}{\underset{︸}{\left(\frac{\partial \rho \tilde{\eta }}{\partial P}+\frac{1}{T}E\right)\cdot \left(\partial_{t}P+\left(\upsilon \cdot \nabla \right)P-\frac{1}{2}\left(\nabla \upsilon -\nabla \upsilon^{T}\right)P\right)}}\\ & +\underset{\xi_{M}}{\underset{︸}{\left(\frac{\partial \rho \tilde{\eta }}{\partial M}+\frac{1}{T}B\right)\cdot \left(\partial_{t}M+\left(\upsilon \cdot \nabla \right)M-\frac{1}{2}\left(\nabla \upsilon -\nabla \upsilon^{T}\right)M\right)}}\;. \end{array}$$

Here, we defined

(56) $$\begin{array}{ll} T= & \sigma +\frac{T}{2}\left(\frac{\partial \rho \tilde{\eta }}{\partial F^{\mathrm{uni}}}F^{\mathrm{uni}}+{\left(F^{\mathrm{uni}}\right)}^{T}{\frac{\partial \rho \tilde{\eta }}{\partial F^{\mathrm{uni}}}}^{T}\right)-\left(\rho u-T\rho \tilde{\eta }-\sum_{\alpha \in I\pm }\rho_{\alpha }\mu_{\alpha }+\frac{T}{3}\mathrm{trace}\left(\frac{\partial \rho \tilde{\eta }}{\partial F^{\mathrm{uni}}}F^{\mathrm{uni}}\right)\right)1\\ & +\frac{1}{2}\left(M⊗B+B⊗M\right)-\frac{1}{2}\left(E⊗P+P⊗E\right)-\left(E\cdot P+M\cdot B\right)1\;, \end{array}$$

(57) $$D=\;\frac{1}{2}\left(\nabla \upsilon +\nabla \upsilon^{T}\right)\;.$$

In the derivation of the entropy production, we used the constraint (18a) to eliminate the flux of the species $A_{0}$, such that only linearly independent mass fluxes appear in the entropy production. Moreover, we used a symmetry condition which originates from the transformation properties of the thermodynamic fields, cf. Appendix B, i.e., we have

(58) $$\frac{\partial \rho \tilde{\eta }}{\partial F_{ik}^{\mathrm{uni}}}F_{jk}^{\mathrm{uni}}+\frac{\partial \rho \tilde{\eta }}{\partial M^{i}}M^{j}+\frac{\partial \rho \tilde{\eta }}{\partial P^{i}}P^{j}=\frac{\partial \rho \tilde{\eta }}{\partial F_{jk}^{\mathrm{uni}}}F_{ik}^{\mathrm{uni}}+\frac{\partial \rho \tilde{\eta }}{\partial M^{j}}M^{i}+\frac{\partial \rho \tilde{\eta }}{\partial P^{j}}P^{i}\;\;\mathrm{for}\;i,j=1,2,3\;.$$

Furthermore, in (56), the binary products of the entropy production are arranged in a way such that the transformation properties required by Axiom III (ii) are guaranteed. The binary product due to shear viscosity is formulated in such a way that the matrices are symmetric. Thus, the anti-symmetric part of (E⊗P)∇υ and (M⊗B)∇υ is shifted to the entropy production of polarization and magnetization, respectively.

To derive constitutive equations from (55), we apply both approaches of Section 5.2. In general, different dissipation mechanism can be coupled, e.g., volume viscosity can be coupled with bulk reactions. To reduce the complexity of the constitutive equations, we only consider cross effects related to thermodiffusion. Since the index sets $I^{\pm }$ are assumed to be disjoint, the closure procedure can be applied in both subdomains independently.

#### Thermodiffusion

For the mass fluxes, we choose a linear relation with cross effects, viz.

(59a) $$q+(E\times M)=\;-\frac{\kappa }{T^{2}}\nabla T-\sum_{\beta \in I^{\pm }\backslash \left\{A_{0\pm }\right\}}L_{\beta }\left(\nabla \left(\frac{\mu_{\beta }}{T}-\frac{\mu_{0}}{T}\right)-\frac{1}{T}\left(\frac{z_{\beta }e_{0}}{m_{\beta }}-\frac{z_{0}e_{0}}{m_{0}}\right)E\right)\;,$$

(59b) $$\begin{array}{l} J_{\alpha }=\;-\frac{L_{\alpha }}{T^{2}}\nabla T-\sum_{\beta \in I^{\pm }\backslash \left\{A_{0\pm }\right\}}M_{\alpha \beta }\left(\nabla \left(\frac{\mu_{\beta }}{T}-\frac{\mu_{0}}{T}\right)-\frac{1}{T}\left(\frac{z_{\beta }e_{0}}{m_{\beta }}-\frac{z_{0}e_{0}}{m_{0}}\right)E\right)\\ \;\alpha \in I^{\pm }\backslash \left\{A_{0\pm }\right\}\;. \end{array}$$

The coefficient matrix $\left(\begin{array}{cc} \kappa & L\\ L^{T} & M \end{array}\right)$ is symmetric and positive definite. In particular, the heat conductivity κ and the mobility matrix *M* are symmetric and positive definite.

#### Reactions

For chemical reactions, often an exponential Arrhenius-type dependence on the temperature and some activation energy is expected. Therefore, we choose the nonlinear relation (53) for the chemical reactions,

(60) $$R^{k}=R_{0}^{k}\left(1-\exp\left(\frac{A^{k}}{k_{B}T}\sum_{\alpha \in I^{\pm }}\gamma_{\alpha }^{k}m_{\alpha }\mu_{\alpha }\right)\right)\;\;\mathrm{for}\;\;k\in \left\{1,\cdots ,M\right\}\;,$$

with positive coefficients $A^{k},R_{0}^{k}$. For simplicity, we neglected cross effects between the different reactions.

#### Viscosity

We choose linear relations for the volume viscosity and for the shear viscosity, viz.

(61a) $$\frac{1}{3}\mathrm{trace}\left(T\right)=\left(\lambda +\frac{2}{3}\eta \right)\mathrm{trace}\left(D\right)\;,$$

(61b) $$T-\frac{1}{3}\mathrm{trace}\left(T\right)1=2\eta \left(D-\frac{1}{3}\mathrm{trace}\left(D\right)1\right)\;,$$

where the phenomenological coefficients satisfy $(\lambda +\frac{2}{3}\eta )>0$ and η>0. The constitutive equations (61) imply a constitutive equation for the symmetric stress tensor σ, viz.

(62) $$\begin{array}{ll} \sigma =- & \frac{T}{2}\left(\frac{\partial \rho \tilde{\eta }}{\partial F^{\mathrm{uni}}}F^{\mathrm{uni}}+{\left(F^{\mathrm{uni}}\right)}^{T}{\frac{\partial \rho \tilde{\eta }}{\partial F^{\mathrm{uni}}}}^{T}\right)+\left(\rho u-T\rho \tilde{\eta }-\sum_{\alpha \in I^{\pm }}\rho_{\alpha }\mu_{\alpha }+\frac{T}{3}\mathrm{trace}\left(\frac{\partial \rho \tilde{\eta }}{\partial F^{\mathrm{uni}}}F^{\mathrm{uni}}\right)\right)1\\ & +\frac{1}{2}\left(E⊗P+P⊗E\right)-\frac{1}{2}\left(M⊗B+B⊗M\right)-\left(E\cdot P-M\cdot B\right)1\\ & +\lambda \mathrm{div}\left(\upsilon \right)1+\eta \left(\nabla \upsilon +{\left(\nabla \upsilon \right)}^{T}\right)\;. \end{array}$$

#### Polarization and Magnetization

For the vector of polarization P and the magnetization M, we choose linear relations without cross effects,

(63a) $$\frac{\tau^{P}}{\varepsilon_{0}}\left(\partial_{t}P+\left(\nabla P\right)\upsilon -\frac{1}{2}\left(\nabla \upsilon -\nabla \upsilon^{T}\right)P\right)=T\frac{\partial \rho \tilde{\eta }}{\partial P}+E\;,$$

(63b) $$\tau^{M}\mu_{0}\left(\partial_{t}M+\left(\nabla M\right)\upsilon -\frac{1}{2}\left(\nabla \upsilon -\nabla \upsilon^{T}\right)M\right)=T\frac{\partial \rho \tilde{\eta }}{\partial M}+B\;.$$

Here, the phenomenological coefficients $\tau^{P}>0$ and $\tau^{M}>0$ are the relaxation times of polarization and magnetization.

### 6.2. Constitutive Relations for the Surface

The exploitation of the entropy principle for the surface is analogous to the bulk. On the surface, we have to determine the heat flux $\underset{s}{q}\;\;$, the mass fluxes $\underset{s}{J}\;{\;}_{\alpha }$, the stress tensor S and the reaction rates $\underset{s}{R}\;{\;}^{k}$. Moreover, we have to determine the normal components of the heat flux $q_{\nu }^{\pm }$, of the mass fluxes $J_{\nu ,\alpha }^{\pm }$ and of the stress tensor $\sigma^{\pm }\cdot \nu$.

First, we substitute the entropy density (48b) into the entropy balance (46a) and choose the entropy flux in a way that allows for the structure (47) of the entropy production, viz.

(64) $$\underset{s}{\phi }\;\;=\frac{\underset{s}{q}\;\;}{\underset{s}{T}\;\;}-\sum_{\alpha \in I_{S}}\frac{\underset{s}{\mu }\;{\;}_{\alpha }}{\underset{s}{T}\;\;}\underset{s}{J}\;{\;}_{\alpha }\;.$$

Then, we identify six dissipation mechanisms: tangential surface viscosity $\underset{s}{\xi }\;{\;}_{V}^{\tau }$, tangential surface thermo-diffusion $\underset{s}{\xi }\;{\;}_{TD}^{\tau }$, surface reactions $\underset{s}{\xi }\;{\;}_{R}$, heat transport normal to the surface $\underset{s}{\xi }\;{\;}_{H}^{\nu }$, mass transport normal to the surface $\underset{s}{\xi }\;{\;}_{MT}^{\nu }$, and viscosity normal to the surface $\underset{s}{\xi }\;{\;}_{V}^{\nu }$. The entropy production on the surface then is

(65) $$\begin{array}{ll} \underset{s}{\xi }\;\;=\; & \frac{1}{\underset{s}{T}\;\;}\left[S^{\Gamma \Delta }+\underset{s}{T}\;\;\frac{\partial \underset{s}{\rho }\;\;\underset{s}{\tilde{\eta }}\;\;}{\partial \tau_{\Delta }^{\mathrm{uni},i}}\tau_{\Sigma }^{\mathrm{uni},i}g^{\Sigma \Gamma }-\left(\underset{s}{\rho }\;\;\underset{s}{u}\;\;-\underset{s}{T}\;\;\underset{s}{\rho }\;\;\underset{s}{\tilde{\eta }}\;\;-\sum_{\alpha \in I_{S}}\underset{s}{\mu }\;{\;}_{\alpha }\underset{s}{\rho }\;{\;}_{\alpha }+\frac{1}{2}\underset{s}{T}\;\;\frac{\partial \underset{s}{\rho }\;\;\underset{s}{\tilde{\eta }}\;\;}{\partial \tau_{\Sigma }^{\mathrm{uni},i}}\tau_{\Sigma }^{\mathrm{uni},i}\right)g^{\Delta \Gamma }\right]\\ & \underset{=\underset{s}{\xi }\;{\;}_{V}^{\tau }}{\underset{︸}{\;\cdot \left(\frac{1}{2}\left(g_{\Gamma \Lambda }\underset{s}{\upsilon }\;{\;}_{\tau ∥\Delta }^{\Lambda }+g_{\Delta \Lambda }\underset{s}{\upsilon }\;{\;}_{\tau ∥\Gamma }^{\Lambda }\right)-b_{\Gamma \Delta }\underset{s}{\upsilon }\;{\;}_{\nu }\right)\;}}\\ & \underset{=\underset{s}{\xi }\;{\;}_{TD}^{\tau }}{\underset{︸}{+\underset{s}{q}\;{\;}^{\Delta }{\left(\frac{1}{\underset{s}{T}\;\;}\right)}_{∥\Delta }-\sum_{\alpha \in I_{S}\backslash \left\{A_{0}\right\}}\underset{s}{J}\;{\;}_{\alpha ,\tau }^{\Delta }\left({\left(\frac{\underset{s}{\mu }\;{\;}_{\alpha }}{\underset{s}{T}\;\;}-\frac{\underset{s}{\mu }\;{\;}_{0}}{\underset{s}{T}\;\;}\right)}_{∥\Delta }-\frac{1}{\underset{s}{T}\;\;}\left(\frac{z_{\alpha }e_{0}}{m_{\alpha }}-\frac{z_{0}e_{0}}{m_{0}}\right)g_{\Delta \Gamma }{\left(\bar{E}+\underset{s}{\upsilon }\;\;\times \bar{B}\right)}_{\tau }^{\Gamma }\right)}}\\ & \underset{\underset{s}{\xi }\;{\;}_{R}}{\underset{︸}{-\frac{1}{\underset{s}{T}\;\;}\sum_{k=1}^{M_{s}}\left(\sum_{\beta \in I_{S}}\underset{s}{\gamma }\;{\;}_{\beta }^{k}m_{\beta }\underset{s}{\mu }\;{\;}_{\beta }\right)\underset{s}{R}\;{\;}^{k}}}\\ & \underset{=\underset{s}{\xi }\;{\;}_{H}^{\nu }}{\underset{︸}{+\left[\;\;\left[\left(q_{\nu }+{\left(E\times M\right)}_{\nu }+\left(T\rho \tilde{\eta }+\sum_{\alpha \in I^{\pm }}\mu_{\alpha }\rho_{\alpha }\right)\left(\upsilon_{\nu }-\underset{s}{\upsilon }\;{\;}_{\nu }\right)\right)\left(\frac{1}{T}-\frac{1}{\underset{s}{T}\;\;}\right)\right]\;\;\right]}}\\ & \underset{=\underset{s}{\xi }\;{\;}_{MT}^{\nu }}{\underset{︸}{-\left[\;\;\left[\sum_{\alpha \in I^{\pm }\backslash \left\{A_{0\pm }\right\}}\left(J_{\alpha ,\nu }+\rho_{\alpha }\left(\upsilon_{\nu }-\underset{s}{\upsilon }\;{\;}_{\nu }\right)\right)\left(\frac{1}{T}\left(\mu_{\alpha }-\mu_{0\pm }\right)-\frac{1}{\underset{s}{T}\;\;}\left(\underset{s}{\mu }\;{\;}_{\alpha }-\underset{s}{\mu }\;{\;}_{0\pm }\right)\right)\right]\;\;\right]}}\\ & +\frac{1}{\underset{s}{T}\;\;}[\;\;[{\left(\upsilon -\underset{s}{\upsilon }\;\;\right)}^{T}(\sigma -E⊗P+M⊗B-\left(\rho u-T\rho \tilde{\eta }-\sum_{\alpha \in I^{\pm }}\mu_{\alpha }\rho_{\alpha }-E\cdot P+M\cdot B\right)1\\ & \underset{=\underset{s}{\xi }\;{\;}_{V}^{\nu }}{\underset{︸}{\;+\left(P\times B\right)⊗\left(\upsilon -\underset{s}{\upsilon }\;\;\right)-\left(\frac{1}{2}\rho {\left|\underset{s}{\upsilon }\;\;-\upsilon \right|}^{2}+\underset{s}{T}\;\;\rho \left(\frac{\mu_{0\pm }}{T}-\frac{\underset{s}{\mu }\;{\;}_{0\pm }}{\underset{s}{T}\;\;}\right)\right)1)\nu ]\;\;]}}\;. \end{array}$$

As in the bulk, the entropy production is formulated such that the factors of the binary products are Galilean scalars, vectors or tensors, respectively. Furthermore, the transformation properties of the thermodynamic fields restrict the entropy density (see Appendix B), and yield the symmetry conditions

(66) $$\frac{\partial \underset{s}{\rho }\;\;\underset{s}{\tilde{\eta }}\;\;}{\partial \tau_{\Delta }^{\mathrm{uni},i}}\tau_{\Sigma }^{\mathrm{uni},i}g^{\Sigma \Gamma }=\frac{\partial \underset{s}{\rho }\;\;\underset{s}{\tilde{\eta }}\;\;}{\partial \tau_{\Gamma }^{\mathrm{uni},i}}\tau_{\Sigma }^{\mathrm{uni},i}g^{\Sigma \Delta }\;\mathrm{for}\;\Gamma ,\Delta =1,2\;\mathrm{and}\;\frac{\partial \underset{s}{\rho }\;\;\underset{s}{\tilde{\eta }}\;\;}{\partial \tau_{\Delta }^{\mathrm{uni},i}}\nu^{i}=0\;\mathrm{for}\;\Delta =1,2\;.$$

The surface entropy production (65) shows some similarities to the bulk entropy production (55), but also some differences. While the bulk entropy production contains two dissipation mechanisms due to polarization and magnetization, there are no similar contributions on the surface because we restricted the constitutive functions $\underset{s}{\rho }\;\;\underset{s}{\tilde{\eta }}\;\;$ on a singular surface to be independent from the electromagnetic fields. Moreover, there are on the surface dissipation mechanisms of different types: while the one class contains only fluxes that are tangential to the surface, the second class consists of those related to the fluxes normal to the surface. In general, it is possible to introduce cross effects between these two different classes, cf. [42]. For simplicity, we do not discuss these kinds of cross effects here.

#### Thermodiffusion

As in the bulk, we chose a linear relation with cross effects between the heat flux $\underset{s}{q}\;\;$ and the mass fluxes $\underset{s}{J}\;{\;}_{\alpha }$,

(67a) $$\begin{array}{ll} \underset{s}{q}\;{\;}_{\tau }^{\Delta }=\; & -\frac{\underset{s}{\kappa }\;\;}{\underset{s}{T}\;{\;}^{2}}g^{\Delta \Gamma }{\left(\underset{s}{T}\;\;\right)}_{∥\Gamma }\\ & \;-\sum_{\beta \in I_{S}\backslash \left\{A_{0}\right\}}\underset{s}{L}\;{\;}_{\beta }\left[g^{\Delta \Gamma }{\left(\frac{\underset{s}{\mu }\;{\;}_{\beta }}{\underset{s}{T}\;\;}-\frac{\underset{s}{\mu }\;{\;}_{0}}{\underset{s}{T}\;\;}\right)}_{∥\Gamma }-\frac{1}{\underset{s}{T}\;\;}\left(\frac{z_{\beta }e_{0}}{m_{\beta }}-\frac{z_{0}e_{0}}{m_{0}}\right){\left(\bar{E}+\underset{s}{\upsilon }\;\;\times \bar{B}\right)}_{\tau }^{\Delta }\right], \end{array}$$

(67b) $$\begin{array}{ll} \underset{s}{J}\;{\;}_{\alpha ,\tau }^{\Delta }=\; & -\frac{\underset{s}{L}\;{\;}_{\alpha }}{\underset{s}{T}\;{\;}^{2}}g^{\Delta \Gamma }{\left(\underset{s}{T}\;\;\right)}_{∥\Gamma }\\ & \;-\sum_{\beta \in I_{S}\backslash \left\{A_{0}\right\}}\underset{s}{M}\;{\;}_{\alpha \beta }\left[g^{\Delta \Gamma }{\left(\frac{\underset{s}{\mu }\;{\;}_{\beta }}{\underset{s}{T}\;\;}-\frac{\underset{s}{\mu }\;{\;}_{0}}{\underset{s}{T}\;\;}\right)}_{∥\Gamma }-\frac{1}{\underset{s}{T}\;\;}\left(\frac{z_{\beta }e_{0}}{m_{\beta }}-\frac{z_{0}e_{0}}{m_{0}}\right){\left(\bar{E}+\underset{s}{\upsilon }\;\;\times \bar{B}\right)}_{\tau }^{\Delta }\right]\;, \end{array}$$

for $\alpha \in I_{S}\backslash \left\{A_{0}\right\}$. The phenomenological matrix $\left(\begin{array}{cc} \underset{s}{\kappa }\;\; & \underset{s}{L}\;\;\\ \underset{s}{L}\;{\;}^{T} & \underset{s}{M}\;\; \end{array}\right)$ is symmetric and positive definite.

#### Surface Reactions

The exponential form of the Butler–Volmer equations for the surface reaction rates, cf. e.g., [10], suggests to apply the nonlinear closure (53). Similar to the bulk and neglecting cross effects between the different reactions, we choose the exponential relations

(68) $$\underset{s}{R}\;{\;}^{k}=\underset{s}{R}\;{\;}_{0}^{k}\left(1-\exp\left(\frac{\underset{s}{A}\;{\;}^{k}}{k_{B}\underset{s}{T}\;\;}\sum_{\alpha \in I_{S}}\underset{s}{\gamma }\;{\;}_{\alpha }^{k}m_{\alpha }\underset{s}{\mu }\;{\;}_{\alpha }\right)\right)\;\;\mathrm{for}\;\;k\in \left\{1,\cdots ,M\right\}\;,$$

with positive phenomenological coefficients $\underset{s}{A}\;{\;}^{k},\underset{s}{R}\;{\;}_{0}^{k}$.

#### Surface Viscosity

Analogously to the bulk, we define the shorthand notation

(69a) $$\underset{s}{T}\;{\;}^{\Delta \Gamma }=S^{\Gamma \Delta }+\underset{s}{T}\;\;\frac{\partial \underset{s}{\rho }\;\;\underset{s}{\tilde{\eta }}\;\;}{\partial \tau_{\Delta }^{\mathrm{uni},i}}\tau_{\Sigma }^{\mathrm{uni},i}g^{\Sigma \Gamma }-\left(\underset{s}{\rho }\;\;\underset{s}{u}\;\;-\underset{s}{T}\;\;\underset{s}{\rho }\;\;\underset{s}{\tilde{\eta }}\;\;-\sum_{\alpha \in I_{S}}\underset{s}{\mu }\;{\;}_{\alpha }\underset{s}{\rho }\;{\;}_{\alpha }+\frac{1}{2}\underset{s}{T}\;\;\frac{\partial \underset{s}{\rho }\;\;\underset{s}{\tilde{\eta }}\;\;}{\partial \tau_{\Sigma }^{\mathrm{uni},i}}\tau_{\Sigma }^{\mathrm{uni},i}\right)g^{\Delta \Gamma }\;,$$

(69b) $$\underset{s}{D}\;{\;}_{\Delta \Gamma }=\frac{1}{2}\left(g_{\Gamma \Lambda }\;\underset{s}{\upsilon }\;{\;}_{\tau ∥\Delta }^{\Lambda }+g_{\Delta \Lambda }\;\underset{s}{\upsilon }\;{\;}_{\tau ∥\Gamma }^{\Lambda }\right)-b_{\Gamma \Delta }\;\underset{s}{\upsilon }\;{\;}_{\nu }\;.$$

The linear closure yields for the trace and for the deviatoric part of the surface stress tensor $\underset{s}{T}\;\;$ the constitutive equations:

(70a) $$\frac{1}{2}\mathrm{trace}\left(\underset{s}{T}\;\;g\right)=\left(\underset{s}{\lambda }\;\;+\underset{s}{\eta }\;\;\right)\mathrm{trace}\left(\underset{s}{D}\;\;\;g^{-1}\right)\;,$$

(70b) $$\underset{s}{T}\;\;-\frac{1}{2}\mathrm{trace}\left(\underset{s}{T}\;\;\;g\right)\;g^{-1}=2\underset{s}{\eta }\;\;\left(g^{-1}\underset{s}{D}\;\;\;g^{-T}-\frac{1}{2}\mathrm{trace}\left(\underset{s}{D}\;\;\;g^{-1}\right)g^{-1}\right)\;.$$

The phenomenological coefficients satisfy $\underset{s}{\lambda }\;\;+\underset{s}{\eta }\;\;\geq 0$ and $\underset{s}{\eta }\;\;\geq 0$. Substituting the definition of $\underset{s}{T}\;\;$ into the constitutive equations (70) yields the constitutive equation for the symmetric surface stress tensor:

(71) $$\begin{array}{ll} S^{\Gamma \Delta }= & -\underset{s}{T}\;\;\frac{\partial \underset{s}{\rho }\;\;\underset{s}{\tilde{\eta }}\;\;}{\partial \tau_{\Delta }^{\mathrm{uni},i}}\tau_{\Sigma }^{\mathrm{uni},i}g^{\Sigma \Gamma }+\left(\underset{s}{\rho }\;\;\underset{s}{u}\;\;-\underset{s}{T}\;\;\underset{s}{\rho }\;\;\underset{s}{\tilde{\eta }}\;\;-\sum_{\alpha \in I_{S}}\underset{s}{\mu }\;{\;}_{\alpha }\underset{s}{\rho }\;{\;}_{\alpha }+\frac{1}{2}\underset{s}{T}\;\;\frac{\partial \underset{s}{\rho }\;\;\underset{s}{\tilde{\eta }}\;\;}{\partial \tau_{\Sigma }^{\mathrm{uni},i}}\tau_{\Sigma }^{\mathrm{uni},i}\right)g^{\Delta \Gamma }\\ & \;+\frac{1}{2}\left(\underset{s}{\lambda }\;\;+\underset{s}{\eta }\;\;\right)\mathrm{trace}\left(\underset{s}{D}\;\;\;g^{-1}\right)+2\underset{s}{\eta }\;\;\left(g^{-1}\underset{s}{D}\;\;\;g^{-T}-\frac{1}{2}\mathrm{trace}\left(\underset{s}{D}\;\;\;g^{-1}\right)g^{-1}\right)\;. \end{array}$$

#### Mass Flux and Stress Normal to the Surface

For the entropy contributions coming from the bulk, we here for simplicity choose the linear relations on *S*. However, we want to mention that the application of the nonlinear closure (53) sometimes can be more appropriate, e.g., in cases where adsorption to the surface becomes the rate limiting step for electron transfer reactions, cf. [17]. We get

(72a) $${\left(q_{\nu }+{\left(E\times M\right)}_{\nu }+\left(T\rho \tilde{\eta }+\sum_{\alpha \in I^{\pm }}\mu_{\alpha }\rho_{\alpha }\right)\left(\upsilon_{\nu }-\underset{s}{\upsilon }\;{\;}_{\nu }\right)\right)}^{\pm }=\pm \underset{s}{\kappa }\;{\;}^{\pm }{\left(\frac{1}{T}-\frac{1}{\underset{s}{T}\;\;}\right)}^{\pm }\;,$$

(72b) $${\left(\sigma^{ij}\nu_{j}g^{\Gamma \Delta }\tau_{\Gamma }^{i}-E_{\tau }^{\Delta }P_{\nu }+M_{\tau }^{\Delta }B_{\nu }+{\left(P\times B\right)}_{\tau }^{\Delta }\left(\upsilon_{\nu }-\underset{s}{\upsilon }\;{\;}_{\nu }\right)\right)}^{\pm }=\pm \underset{s}{\eta }\;{\;}^{\pm }{\left(\upsilon_{\tau }^{\Delta }-\underset{s}{\upsilon }\;{\;}_{\tau }^{\Delta }\right)}^{\pm }\;,$$

(72c) $$\begin{array}{r} {\left(J_{\alpha ,\nu }+\rho_{\alpha }\left(\upsilon_{\nu }-\underset{s}{\upsilon }\;{\;}_{\nu }\right)\right)}^{\pm }=\mp \underset{s}{M}\;{\;}_{\alpha }^{\pm }{\left(\frac{1}{T}\left(\mu_{\alpha }-\mu_{0\pm }\right)-\frac{1}{\underset{s}{T}\;\;}\left(\underset{s}{\mu }\;{\;}_{\alpha }-\underset{s}{\mu }\;{\;}_{0\pm }\right)\right)}^{\pm }\\ \mathrm{for}\;\alpha \in I^{\pm }\backslash \left\{A_{0\pm }\right\}\;. \end{array}$$

(72d) $$\begin{array}{r} (\nu^{T}\left(\sigma -E⊗P+M⊗B\right)\nu -\left(\rho u-T\rho \tilde{\eta }-\sum_{\alpha \in I^{\pm }}\mu_{\alpha }\rho_{\alpha }-E^{k}P^{k}+M^{k}B^{k}\right)\;\\ +{\left(P\times B\right)}_{\nu }\left(\upsilon_{\nu }-\underset{s}{\upsilon }\;{\;}_{\nu }\right)-\left(\frac{1}{2}\rho {\left|\underset{s}{\upsilon }\;\;-\upsilon \right|}^{2}+\underset{s}{T}\;\;\rho \left(\frac{\mu_{0\pm }}{T}-\frac{\underset{s}{\mu }\;{\;}_{0\pm }}{\underset{s}{T}\;\;}\right)\right){)}^{\pm }\\ =\pm \underset{s}{\lambda }\;{\;}^{\pm }\rho^{\pm }{\left(\rho (\upsilon_{\nu }-\underset{s}{\upsilon }\;{\;}_{\nu })\right)}^{\pm }\;. \end{array}$$

The coefficients $\underset{s}{\kappa }\;{\;}^{\pm }$, $\underset{s}{\eta }\;{\;}^{\pm }$ and $\underset{s}{\lambda }\;{\;}^{\pm }$ are positive and the matrices $\underset{s}{M}\;{\;}^{\pm }$ are symmetric and positive definite.

### 6.3. Remarks on the Constitutive Relations

#### Comparison of Bulk and Surface Equations

Comparison of the constitutive equations for bulk and surface reveals that, in the case of vanishing polarization and magnetization, the bulk and surface equations have an analogous mathematical structure. The differences are due to the fact that we do not consider surface polarization and surface magnetization in constitutive equation (48b) for the surface entropy density. For vanishing polarization and magnetization, the electromagnetic field solely contributes to the mass flux and heat flux in the form of the electromotive intensity E. Although a contribution of the electromagnetic field to the surface mass fluxes (67b), which results from the linear closure we applied here, has to be expected due to the symmetry properties between bulk and surface, it is not present in literature [21,22,43].

#### Free Energy Density

The temperature *T* is a quantity of central interest in thermodynamics. It is here defined as the derivative of the entropy density with respect to the internal energy density. For the construction of constitutive equations, it is often beneficial to use the temperature *T* resp. $\underset{s}{T}\;\;$ as an independent variable, instead of the internal energy density. To replace the internal energy density as an independent variable by the temperature, the free energy density ρψ is introduced by means of Legendre transformation of the entropy density ρη, viz.

(73) $$\rho \psi =\rho u+M\cdot B-T\;\rho \eta \;,\;\;\underset{s}{\rho }\;\;\underset{s}{\psi }\;\;=\underset{s}{\rho }\;\;\underset{s}{u}\;\;-\underset{s}{T}\;\;\;\underset{s}{\rho }\;\;\underset{s}{\eta }\;\;\;.$$

For the free energy density, we thus have representations as functions of the temperature

(74) $$\rho \psi =\rho \hat{\psi }\left(T,{\left(\rho_{\alpha }\right)}_{\alpha \in I^{\pm }},F^{\mathrm{uni}},P,M\right)\;,\;\underset{s}{\rho }\;\;\underset{s}{\psi }\;\;=\underset{s}{\rho }\;\;\underset{s}{\hat{\psi }}\;\;\left(\underset{s}{T}\;\;,\left(\underset{s}{\rho }\;{\;}_{\alpha }\right){}_{\alpha \in I_{S}},\tau_{1}^{\mathrm{uni}},\tau_{2}^{\mathrm{uni}}\right)\;.$$

Moreover, the Legendre transformation implies relations between the constitutive functions of the free energy density and the entropy density. For $\rho \hat{\eta }$ and $\underset{s}{\rho }\;\;\underset{s}{\hat{\eta }}\;\;$ denoting the constitutive functions depending on the temperature *T* respective $\underset{s}{T}\;\;$, we obtain

(75a) $$\frac{\partial \rho \hat{\psi }}{\partial T}=-\rho \hat{\eta }\;\mathrm{and}\;\frac{\partial \rho \hat{\psi }}{\partial X}=-\frac{1}{T}\frac{\partial \rho \hat{\eta }}{\partial X}\;\mathrm{for}\;X\in {\left\{\rho_{\alpha }\right\}}_{\alpha \in I^{\pm }}\cup \left\{F^{\mathrm{uni}},P,M\right\}\;,$$

(75b) $$\frac{\partial \underset{s}{\rho }\;\;\underset{s}{\hat{\psi }}\;\;}{\partial \underset{s}{T}\;\;}=-\underset{s}{\rho }\;\;\underset{s}{\hat{\eta }}\;\;\;\mathrm{and}\;\frac{\partial \underset{s}{\rho }\;\;\underset{s}{\hat{\psi }}\;\;}{\partial \underset{s}{X}\;\;}=-\frac{1}{\underset{s}{T}\;\;}\frac{\partial \underset{s}{\rho }\;\;\underset{s}{\hat{\eta }}\;\;}{\partial \underset{s}{X}\;\;}\;\mathrm{for}\;\underset{s}{X}\;\;\in \left\{\underset{s}{\rho }\;{\;}_{\alpha }\right\}{}_{\alpha \in I_{S}}\cup \left\{\tau_{1}^{\mathrm{uni}},\tau_{2}^{\mathrm{uni}}\right\}\;.$$

From the definition (73) and using the transformation properties (75), we get relations between the internal energy density and the free energy density:

(76) $$\frac{\partial }{\partial T}\left(\frac{\rho \hat{\psi }}{T}\right)=-\frac{\rho \hat{u}+M\cdot B}{T^{2}}\;,\;\;\frac{\partial }{\partial \underset{s}{T}\;\;}\left(\frac{\underset{s}{\rho }\;\;\underset{s}{\hat{\psi }}\;\;}{\underset{s}{T}\;\;}\right)=-\frac{\underset{s}{\rho }\;\;\underset{s}{\hat{u}}\;\;}{\underset{s}{T}\;{\;}^{2}}\;.$$

#### Pressure, Surface Tension and Gibbs–Duhem Equation

Further important thermodynamic quantities are the pressure *p* and the surface tension $\underset{s}{\gamma }\;\;$. Their definition is not unique and different approaches can be found in the literature, in particular in the presence of electromagnetic fields. Here, we define pressure and surface tension as the traces of the stress tensors, i.e.,

(77) $$p=-\frac{1}{3}\mathrm{trace}\left(\sigma \right)\;,\;\;\underset{s}{\gamma }\;\;=\frac{1}{2}\mathrm{trace}\left(S\;g\right)\;.$$

The constitutive equations (62) and (71) then imply that pressure and surface tension depend on deformation, viscosity, polarization and magnetization.

In the case of vanishing electromagnetic fields and vanishing viscosity, the constitutive equations for pressure and surface tension simplify to

(78) $$p=-\rho \psi +\sum_{\alpha \in I^{\pm }}\rho_{\alpha }\mu_{\alpha }\;,\;\;\underset{s}{\gamma }\;\;=\underset{s}{\rho }\;\;\underset{s}{\psi }\;\;-\sum_{\alpha \in I_{S}}\underset{s}{\rho }\;{\;}_{\alpha }\underset{s}{\mu }\;{\;}_{\alpha }\;.$$

These equations are the well known Gibbs–Duhem relation and its counterpart on the surface, cf. [20,21]. If the velocities can be neglected, i.e., υ=0 and $\underset{s}{\upsilon }\;\;=0$, then the normal component of the surface momentum balance (22b) simplifies to the Young–Laplace equation

(79) $$\left[\;\left[p\right]\;\right]=2\underset{s}{\gamma }\;\;k_{M}\;.$$

#### Adsorption

In chemistry, an adsorption process is often described as a reaction that contributes to the mass production $\underset{s}{r}\;{\;}_{\alpha }$ for $\alpha \in I_{S}$. A similar approach is not possible in the context of electrothermodynamics because the constitutive equations for the surface reactions cannot directly couple bulk and surface species. Thus, adsorption of species is here determined by the constitutive equations (72d) and (72c) for the mass fluxes in normal direction onto the surface. The driving forces for the mass fluxes are the differences of chemical potentials between bulk and surface. The adsorption rate of a species $A_{\alpha }$ is thus determined by a kinetic coefficient $\underset{s}{M}\;{\;}_{\alpha }^{\pm }$ in Equation (72c). If species $A_{\alpha }$ is non-adsorbing, then $\underset{s}{M}\;{\;}_{\alpha }^{\pm }=0$.

In addition, the total mass flux $\rho {\left(\upsilon_{\nu }-\underset{s}{\upsilon }\;{\;}_{\nu }\right)}^{\pm }$ in normal direction onto the surface is determined by the constitutive equation (72d). In particular, it depends on the Cauchy stress tensor. Under the assumption that the viscosity in the bulk is small and the free energy density in the bulk is independent from the deformation gradient, i.e., $\rho \psi =\rho \hat{\psi }\left(T,{\left(\rho_{\alpha }\right)}_{\alpha \in I^{\pm }},P,M\right)$, then relation (72d) simplifies to (due to constitutive equation (62) for σ)

(80) $${\left({\left(P\times B\right)}_{\nu }\left(\upsilon_{\nu }-\underset{s}{\upsilon }\;{\;}_{\nu }\right)-\left(\frac{1}{2}\rho {\left|\underset{s}{\upsilon }\;\;-\upsilon \right|}^{2}+\underset{s}{T}\;\;\rho \left(\frac{\mu_{0}}{T}-\frac{\underset{s}{\mu }\;{\;}_{0}}{\underset{s}{T}\;\;}\right)\right)\right)}^{\pm }=\pm \underset{s}{\lambda }\;{\;}^{\pm }\rho^{\pm }{\left(\rho (\upsilon_{\nu }-\underset{s}{\upsilon }\;{\;}_{\nu })\right)}^{\pm }\;.$$

If also the electromagnetic contribution ${\left(P\times B\right)}_{\nu }\left(\upsilon_{\nu }-\underset{s}{\upsilon }\;{\;}_{\nu }\right)$ as well as kinetic contribution $\frac{1}{2}\rho {\left|\underset{s}{\upsilon }\;\;-\upsilon \right|}^{2}$ vanishes, then the total mass flux is determined by the adsorption of species $A_{0}$.

### 6.4. Discussion on Polarization and Debye Equation for Dielectric Relaxation

The constitutive equations of this section are consequences of the choice of the variables for the entropy density (48a). In addition, various different sets of variables can be compatible with the axioms I–III of the entropy principle as well. In particular, the choice of P and M is not mandatory, and it might seem more natural to use E and B instead.

In local equilibrium, i.e., $\xi_{P}=0$ and $\xi_{M}=0$ in the entropy production (55), there is no preference for either choice. The constitutive equations (63) then imply

(81) $$E=\frac{\partial \rho \tilde{\psi }}{\partial P}\;,\;\;B=\frac{\partial \rho \tilde{\psi }}{\partial M}\;.$$

By the Legendre transformation, the variables (P, M) can be replaced by (E, B), cf. e.g., the discussion of the different sets of variables in [31].

#### Relaxation

The situation changes decisively in non-equilibrium. For simplicity, let us consider a system with only one species and two different constitutive functions for the entropy density

(82) $$\rho \eta =\rho {\tilde{\eta }}^{PM}\left(\rho u+M\cdot B,\rho ,P,M\right)\;,\;\;\rho \eta =\rho {\tilde{\eta }}^{EB}\left(\rho u-E\cdot P,\rho ,E,B\right)\;.$$

In each setting, an according temperature is then defined as

(83) $$\frac{1}{T^{PM}}=\frac{\partial \rho {\tilde{\eta }}^{PM}}{\partial \rho u+M\cdot B}\;,\;\;\frac{1}{T^{EB}}=\frac{\partial \rho {\tilde{\eta }}^{EB}}{\partial \rho u-E\cdot P}\;.$$

The corresponding free energy densities that follow from (82) and (83) are

(84) $$\rho \psi^{PM}:=\rho u-T^{PM}\rho {\tilde{\eta }}^{PM}+M\cdot B\;,\;\;\rho \psi^{EB}:=\rho u-T^{EB}\rho {\tilde{\eta }}^{EB}-P\cdot E\;.$$

This coincides with the cases (a) and case (c) in ([31], Section 3.3) if the internal energy density is defined as ρu+M·B, as we did in axiom IV of the entropy principle. The corresponding constitutive functions of the free energy densities are

(85) $$\rho \psi^{PM}=\rho {\hat{\psi }}^{PM}\left(T^{PM},\rho ,P,M\right)\;,\;\;\rho \psi^{EB}=\rho {\hat{\psi }}^{EB}\left(T^{EB},\rho ,E,B\right)\;.$$

Then, exploitation of the entropy principle yields for the two cases the respective entropy productions due to polarization

(86a) $$\xi_{P}^{PM}=+\frac{1}{T^{PM}}\left(E-\frac{\partial \rho {\hat{\psi }}^{PM}}{\partial P}\right)\left(\partial_{t}P+\nabla P\;\upsilon -\frac{1}{2}P\left(\nabla \upsilon -\nabla \upsilon^{T}\right)\right),$$

(86b) $$\xi_{P}^{EB}=-\frac{1}{T^{EB}}\left(P+\frac{\partial \rho {\hat{\psi }}^{EB}}{\partial E}\right)\left(\partial_{t}E+\nabla E\;\upsilon -\frac{1}{2}E\left(\nabla \upsilon -\nabla \upsilon^{T}\right)\right).$$

Both $\xi_{P}^{PM}$ and $\xi_{P}^{EB}$, are compatible with the structure of the entropy production (47) in the axiom III(ii). Comparing Equations (86a) and (86b), we observe that upon an interchange of the variables P and E the two relations are almost identical. However, while the entropy production $\xi_{P}^{PM}$ begins with a positive sign, there is a minus in $\xi_{P}^{EB}$.

For further analysis, it is necessary to specify the dependence of free energy density on (P, M) respective (E, B). We consider the most simple choice

(87) $$\rho \psi^{PM}=\rho {\hat{\hat{\psi }}}^{PM}\left(T^{PM},\rho ,M\right)+\frac{1}{2\varepsilon_{0}\chi }{\left|P\right|}^{2}\;,\rho \psi^{EB}=\rho {\hat{\hat{\psi }}}^{EB}\left(T^{EB},\rho ,B\right)-\frac{\varepsilon_{0}}{2}\chi {\left|E\right|}^{2}\;,$$

with a constant electric susceptibility χ. When now applying the linear closure to derive the constitutive equations, we get in the case of vanishing velocity, i.e., υ=0 the constitutive equations

(88a) $$\frac{1}{\varepsilon_{0}}\tau^{PM}\partial_{t}P=+\left(E-\frac{1}{\varepsilon_{0}\chi }P\right)\;,$$

(88b) $$\varepsilon_{0}\tau^{EB}\partial_{t}E=-\left(P-\varepsilon_{0}\chi E\right),$$

where the relaxation constants $\tau^{PM},\tau^{EB}>0$ are positive. We conclude from the constitutive equation (63a)

(89) $$E=\frac{\partial \rho \psi^{PM}}{\partial P}\;\;\mathrm{and}\;\;P=-\frac{\partial \rho \psi^{EB}}{\partial E}\;\;\mathrm{in}\;\mathrm{equilibrium},$$

such that in both cases the polarization is proportional to the electromotive intensity, i.e.,

(90) $$P=\varepsilon_{0}\chi E\;\;\mathrm{in}\;\mathrm{equilibrium}.$$

In non-equilibrium, the situation is different. For simplicity, let us assume that the magnetization is in local equilibrium, i.e., $\xi_{M}=0$, and the free energy densities (85) are independent from M and B respectively. Under this simplification M=0, B=0 and E=E, and Maxwell’s equations simplify to

(91) $$\mathrm{div}(\varepsilon_{0}E+P)=0\;,\mathrm{curl}E=0\;.$$

Then, the divergence of the constitutive equations (88) simplify to

(92a) $$(88a)\;\&\;(91)⟹\;\tau^{PM}\partial_{t}\mathrm{div}\left(P\right)=-\left(1+\frac{1}{\chi }\right)\mathrm{div}\left(P\right)\;,$$

(92b) $$(88b)\;\&\;(91)⟹\;\tau^{EB}\partial_{t}\mathrm{div}\left(E\right)=+(1+\chi )\mathrm{div}(E)\;.$$

Now we see that, in Equation (92a), div(P) vanishes for t→∞. In contrast, Equation (92b) implies that div(E) blows up in time with an exponential growth. We conclude that, although both choices of independent variables, (P, M) and (E, B), yield the same equilibrium relation (90), and in both cases the entropy production is non-negative due to the linear closure (88), the latter choice of the relaxation equation (92b) is incompatible with the equilibrium relations. Therefore, the approach based on the independent variables P, M should be strongly preferred. An analogous argumentation also holds for the magnetization.

We may recapitulate the described problem by stating: the entropy principle does not necessarily prevent instability of the resulting system of field equations. This phenomenon is already known in a different area. Non-Newtonian fluids can be modeled with differential type constitutive functions for the stress, such that the symmetric part of the velocity gradient and its time derivatives are among the variables. This approach leads likewise to exponential growth in a situation where the fluid is expected to approach equilibrium. The problem is removed by interchanging the role of the velocity gradient and the stress as variable and constitutive quantity. The details are carefully described in [44,45].

#### Debye Equation

The Debye equation is used to describe for ideal systems the dielectric relaxation, i.e., the response of the electric field inside a dielectric material to the excitation by an oscillatory outer electric field, cf. e.g., [20,46]. In our setting, the Debye equation is given by Equation (88a) and its derivation is an immediate consequence of the entropy principle of Section 5.2 and it is identical to the Debye equation found by deGroot and Mazur in ([20], p. 400) for matter at rest. In the case of non-vanishing velocities, i.e., υ≠0, the Debye equation found by deGroot and Mazur is not identical to Equation (63a) in this paper.

## 7. Application to Electrochemical Systems

The constitutive equations of Section 6 have been derived without making use of any particular specific material properties. They only rely on the universal balance equations and the entropy principle. All material properties of a specific electrochemical system thus have to be incorporated into the constitutive functions of the entropy and the phenomenological coefficients. In this section, we illustrate the application of the developed theoretical framework for the example of liquid electrolytes in contact with metal electrodes.

Our main interest is the accurate description of the charge transport in electrolytes and electrochemical processes at the electrode–electrolyte interface. By interface, we always mean the compound consisting of surface and the adjacent boundary layers from both sides. We summarize recent results [14,15,16,17,18,19], which are related to an improved understanding of the double layer structure that in particular allows quantitative and qualitative prediction of the differential double layer capacity, and enables a better understanding of electrocapillarity effects. Moreover, the above developed framework allows the formulation of extended Nernst–Planck fluxes and constitutive equations for surface electron transfer reactions that allow to recover generalized Butler–Volmer equations.

A particular difficulty for the development of mathematical models for batteries, fuel cells, electrolyzers or other electrochemical systems is due to the complexity that results from very different scales in space and time, the coupling of bulk and surface processes and the interplay between different physical phenomena like electrical and mechanical phenomena. Therefore, we first identify simplifying assumptions that are appropriate to adapt the most general theory developed above to the description of liquid electrolytes and metal–electrolyte interfaces.

### 7.1. Dimensional Analysis of Maxwell’s Equations

To write Maxwell’s equations in non-dimensional form, we introduce characteristic reference values $t^{\mathrm{ref}},x^{\mathrm{ref}}$ for the time and space coordinates, $n_{\alpha }^{\mathrm{ref}}$ for the number densities, and $E^{\mathrm{ref}},B^{\mathrm{ref}}$ for the electromagnetic field. The velocity, polarization and magnetization are scaled by reference values derived from those above, i.e., $v^{\mathrm{ref}}=x^{\mathrm{ref}}/t^{\mathrm{ref}}$, $P^{\mathrm{ref}}=\varepsilon_{0}E^{\mathrm{ref}}$, $M^{\mathrm{ref}}=\frac{1}{\mu_{0}}B^{\mathrm{ref}}$. Upon introduction of the dimensionless quantities

(93) $$\lambda =\sqrt{\frac{\varepsilon_{0}E^{\mathrm{ref}}}{e_{0}n^{\mathrm{ref}}x^{\mathrm{ref}}}}\;,\beta =\sqrt{\frac{B^{\mathrm{ref}}v^{\mathrm{ref}}}{E^{\mathrm{ref}}}}\;,\delta =\beta \lambda \frac{c_{0}}{\upsilon^{\mathrm{ref}}}\;,$$

we get the following dimension less form of Maxwell’s equations in the bulk

(94a) $$\beta^{2}\frac{\partial \overset{˘}{B}}{\partial t}+\mathrm{curl}\overset{˘}{E}=0\;,$$

(94b) $$\mathrm{div}\overset{˘}{B}=0\;,$$

(94c) $$-\lambda^{2}\frac{\partial (\overset{˘}{E}+\overset{˘}{P})}{\partial t}+\delta^{2}\mathrm{curl}\left(\overset{˘}{B}-\overset{˘}{M}\right)={\overset{˘}{n}}^{F}\overset{˘}{\upsilon }+{\overset{˘}{J}}^{F}\;,$$

(94d) $$\lambda^{2}\mathrm{div}\left(\overset{˘}{E}+\overset{˘}{P}\right)={\overset{˘}{n}}^{F}\;.$$

The non-dimensional bulk definitions of electromotive intensity and magnetization read

(95) $$\overset{˘}{E}=\overset{˘}{E}+\beta^{2}\left(\overset{˘}{\upsilon }\times \overset{˘}{B}\right)\;,\overset{˘}{M}=\overset{˘}{M}+\frac{\lambda^{2}}{\delta^{2}}\left(\overset{˘}{\upsilon }\times \overset{˘}{P}\right)\;.$$

Depending on the chosen characteristic reference values, the size of the dimensionless quantities may differ by several orders of magnitude, allowing considerable simplifications of the system.

#### Magnetostatics: λ→0

Under the assumption that the derivatives of $\overset{˘}{E}+\overset{˘}{P}$ remain bounded, Maxwell’s equations simplify in the asymptotic limit λ→0. Rescaled to dimensional quantities, we get the equations of magnetostatics for the magnetic flux density B,

(96a) divB=0,

(96b) $$\mathrm{curl}(\frac{1}{\mu_{0}}B-M)=J^{F}\;,$$

and the magnetization is identical to the Lorentz magnetization, i.e., M=M. The two remaining Maxwell’s equations are

(97) $$\frac{\partial B}{\partial t}+\mathrm{curl}E=0\;,0=n^{F}\;,$$

which, in conjunction with the equation of matter, determine the electric field E.

#### Electrostatics: β,1/δ→0

In this limit, the equations of electrostatics for the electric field are in dimensional form

(98a) curlE=0,

(98b) $$\mathrm{div}(\varepsilon_{0}E+P)=n^{F}\;,$$

and the electromotive intensity is identical to the electric field, i.e., E=E. The remaining Maxwell’s equations determine the magnetic flux density B, i.e.,

(99) $$\mathrm{div}B=0\;,\mathrm{curl}(\frac{1}{\mu_{0}}B-M)=0\;.$$

Equation (98a) implies the existence of an *electrostatic potential* φ, such that

(100) E=−∇φ.

Assuming a simple constitutive equation for the polarization, i.e., $P=\chi \varepsilon_{0}E$, where χ is the electric susceptibility, we get from Equation (98b) Poisson’s equation for the electrostatic potential, viz.

(101) $$-\left(1+\chi \right)\varepsilon_{0}\Delta \varphi =n^{F}\;.$$

The dimensional analysis can be performed in analogue manner for the surface equations, cf. [13]. In the limit β→0 and 1/δ→0, Maxwell’s surface equations yield two boundary conditions for the electrostatic potential,

(102) $$\underset{s}{n}\;{\;}^{F}=-\varepsilon_{0}\left[\;\left[\left(1+\chi \right)\nabla \varphi \right]\;\right]\;,0=\nu \times [\;[\nabla \varphi ]\;]\;,$$

where the susceptibility χ can have different values on both sides of the surface. Due to the second condition in Equation (102), the jump of the potential [[φ]] is determined up to a constant and, in particular, this constant is independent from the material. Since the equations for the electrostatic potential allow a normalization, we can set this constant to zero. Thus, the electrostatic potential is continuous at the surface, which allows for defining the electrostatic potential of the surface as

(103) $$\underset{s}{\varphi }\;\;=\varphi {{|}_{S}^{+}=\varphi |}_{S}^{-}\;\mathrm{on}\;S\;.$$

#### Application to Electrochemical Systems

We consider a system that is varying moderately in time, has a number density in a typical range for solids and liquids, and a magnetic field strength that does not significantly exceed the field of common permanent magnets, viz.

(104) $$t^{\mathrm{ref}}=1s\;,n^{\mathrm{ref}}=10^{28}m^{-3}\;,B^{\mathrm{ref}}=10^{-1}\frac{As}{m^{2}}\;.$$

An important feature of electrochemical systems is the formation of narrow double layers at the contact of different materials. The double layer is characterized by a typical width in the range of nanometers and a strong electric field in the range of $1\frac{V}{\mathrm{nm}}$. We thus choose the reference values

(105) $$x^{\mathrm{ref}}=10^{-9}\;m\;,E^{\mathrm{ref}}=10^{9}\frac{V}{m}\;,$$

implying for the dimensionless quantities

(106) $$\lambda^{2}\approx 10^{-2}\;,\beta^{2}\approx 10^{-19}\;,\delta^{2}\approx 10^{14}\;.$$

The smallness of $\beta^{2}$ and $1/\delta^{2}$ compared to $\lambda^{2}$ suggests for the determination of E the use of the electrostatic limit equations (98), or (101) and (102), respectively. In conclusion, for electrochemical systems, the electrostatic approximation is valid, as long as the characteristic values of the system at hand are in the order of magnitude defined in Equations (104) and (105). In particular, in systems where the characteristic time scale is smaller, for instance for impedance measurements, the application of the electrostatic approximation is not appropriate.

### 7.2. Free Energy Models for Liquid Electrolytes and Metal–Electrolyte Interfaces

To develop a complete mathematical model for liquid electrolytes and metal–electrolyte interfaces, we start with some simplifying assumptions. The metal can be described by applying the Sommerfeld free electron model [47]. Then, it turns out that most of the relevant information of the metal–electrolyte interface can already be obtained without solving the model equations on the metal side, cf. [18]. Thus, we here ignore the metal electrode and only consider the electrolyte subdomain that further on is denoted by Ω with the corresponding index set I. For the description of polarizable liquid electrolytes, it is reasonable to neglect the bulk and surface deformation gradients $F^{\mathrm{uni}}$ and $\tau_{1/2}^{\mathrm{uni}}$ and, in particular based on the previous dimensional analysis, the dependency of the free energy function on the magnetization M. The constitutive function for the free energy densities simplify to

(107) $$\rho \psi =\rho \hat{\psi }\left(T,{\left(\rho_{\alpha }\right)}_{\alpha \in I},P\right)\;,\underset{s}{\rho }\;\;\underset{s}{\psi }\;\;=\underset{s}{\rho }\;\;\underset{s}{\hat{\psi }}\;\;\left(\underset{s}{T}\;\;,\left(\underset{s}{\rho }\;{\;}_{\alpha }\right){}_{\alpha \in I_{S}}\right)\;.$$

We consider isothermal processes only. Thus, the temperature in the bulk and on surface is given by an appropriate reference temperature $T^{\mathrm{ref}}$ that usually be the room temperature:

(108) $$T=\underset{s}{T}\;\;=T^{\mathrm{ref}}\;.$$

The energy balances (25) then only serve to determine the heat fluxes which are necessary to enable an isothermal process.

In the following, free energy functions for bulk and surface are developed. We only sketch the main ideas of the derivation and refer for more details to [13,14,15,18]. In particular, we illustrate here how elasticity contributes to the free energy function and how finite ion sizes can be incorporated into the electrolyte model.

#### Bulk Free Energy Density

The free energy density is not directly measurable. Therefore, a derivation of a free energy density must be based on constitutive relations that are in simple equilibrium situations backed by sufficient empirical evidence or that are derived from a microscopic theory. For liquid electrolytes, we consider free energy functions which consist of four contributions that are related to polarization, mechanical stresses, mixing entropy and a temperature dependent reference contribution, i.e.,

(109) $$\rho \psi =\rho \psi^{\mathrm{pol}}+\rho \psi^{\mathrm{mech}}+\rho \psi^{\mathrm{mix}}+\rho \psi^{\mathrm{ref}}\;.$$

For the derivation of the individual contributions of the free energy, it is convenient to introduce the total mole density of particles *n* and the mole fractions $y_{\alpha }$ by

(110) $$n=\sum_{\alpha \in I}n_{\alpha }\;\mathrm{and}\;y_{\alpha }=\frac{n_{\alpha }}{n}\;\mathrm{with}\;\sum_{\alpha \in I}y_{\alpha }=1\;.$$

The entropy of mixing accounts for the number of possible arrangements of ions and solvent molecules for a give macroscopic state. It is determined by statistical thermodynamics by means of the Boltzmann formula. Thus, the corresponding free energy contribution reads

(111) $$\rho \psi^{\mathrm{mix}}\left(T,{\left(\rho_{\alpha }\right)}_{\alpha \in I}\right)=k_{B}T\sum_{\alpha \in I}n_{\alpha }\ln\left(y_{\alpha }\right)\;.$$

The mechanical part of the free energy density function is derived by integration of the identity $p=\rho^{2}\frac{\partial }{\partial \rho }\left(\frac{\rho \hat{\psi }}{\rho }\right)$, which in turn is derived from a generalization of the Gibbs–Duhem equation (78), cf. [13,48]. Equation (78) is well known for non-polarizable materials. Since the polarization (116) is independent from the number densities, the definition of the Gibbs–Duhem equation (78) can be easily extended to the case handled here (see [14,15]). We assume a simple linear constitutive relation for the pressure *p*,

(112) $$p=p^{R}+K\left(nH-1\right)\;\mathrm{with}\;H=\sum_{\alpha \in I}v_{\alpha }^{\mathrm{ref}}y_{\alpha }\;.$$

Herein, $p^{\mathrm{ref}}$ is the pressure of the reference state and *K* denotes the bulk modulus of the electrolyte. By $v_{\alpha }^{\mathrm{ref}}$, we denote the partial specific volume of the constituent $A_{\alpha }$ under the pressure $p^{\mathrm{ref}}$ and temperature $T^{\mathrm{ref}}$. The function *H* is the mean specific volume of the mixture and accounts for volume changes due to a local variation of the mixtures composition. The mechanical contribution to the free energy density is derived from the constitutive equation (112) as

(113) $$\rho \psi^{\mathrm{mech}}\left(T,{\left(\rho_{\alpha }\right)}_{\alpha \in I}\right)=\left(p^{\mathrm{ref}}-K\right)\left(n\;H-1\right)+K\;n\;H\ln\left(nH\right)\;.$$

The reference contribution to the free energy is assumed to be

(114) $$\rho \psi^{\mathrm{ref}}\left(T,{\left(\rho_{\alpha }\right)}_{\alpha \in I}\right)=\sum_{\alpha \in I}\rho_{\alpha }\psi_{\alpha }^{\mathrm{ref}},$$

where $\psi_{\alpha }^{\mathrm{ref}}$ denotes the reference free energy of each individual constituent. In the reference values $\psi_{\alpha }^{\mathrm{ref}}$, the specific heat is encoded, but this is not further outlined here because of the assumption of isothermal processes.

In equilibrium, we want to maintain the simple constitutive relation between electric field and polariziation

(115) $$P=\chi \varepsilon_{0}E\;.$$

An integration of relation (63a) in equilibrium and in the electrostatic approximation, i.e., $0\;=-\frac{\partial \rho \psi }{\partial P}+E$ then yields the corresponding contribution to the free energy density due to polarization

(116) $$\rho \psi^{\mathrm{pol}}\left(T,P\right)=\frac{1}{2\varepsilon_{0}\chi }\left|P^{2}\right|\;.$$

Here, the susceptibility χ is assumed to be independent from the number densities, but may be dependent on the temperature. The assumption that the susceptibility is independent from number densities is not necessary, but this leads to simplified constitutive relations, in particular the chemical potentials are independent from polarization.

From the free energy contributions (109)–(114) and the definition (49a), we get the chemical potentials

(117) $$\mu_{\alpha }=\frac{1}{m_{\alpha }}\psi_{\alpha }^{\mathrm{ref}}+\frac{v_{\alpha }^{\mathrm{ref}}}{m_{\alpha }}\left(p^{\mathrm{ref}}+K\;\ln\left(1+\frac{p-p^{\mathrm{ref}}}{K}\right)\right)+\frac{k_{B}T}{m_{\alpha }}\ln y_{\alpha }\;\mathrm{for}\;\alpha \in I\;.$$

Here, we used the constitutive relation (112) to express the pressure dependency of the chemical potentials.

#### Surface Free Energy Density

A simple surface free energy model of a metal–electrolyte interface was proposed in [18] that we will summarize here. On the surface, there is no contribution from the electric field, but, as in the volume, we assume additive mechanical, entropic and reference contributions, i.e.,

(118) $$\underset{s}{\rho }\;\;\underset{s}{\psi }\;\;=\underset{s}{\rho }\;\;\underset{s}{\psi }\;{\;}^{\mathrm{mech}}+\underset{s}{\rho }\;\;\underset{s}{\psi }\;{\;}^{\mathrm{mix}}+\underset{s}{\rho }\;\;\underset{s}{\psi }\;{\;}^{\mathrm{ref}}\;,$$

where the reference contributions are of the form

(119) $$\underset{s}{\rho }\;\;\underset{s}{\psi }\;{\;}^{\mathrm{ref}}=\sum_{\alpha \in I_{S}}\underset{s}{\rho }\;{\;}_{\alpha }\underset{s}{\psi }\;{\;}_{\alpha }^{\mathrm{ref}}\;.$$

The reference values may depend on the temperature and in general also on the crystallographic orientation of the surface. The set of constituents $I_{S}$ on the surfaces contains all electrolytic bulk species, the metal bulk constituents, which are the electrons e and metal ions M, and in addition a certain number of reaction products of the bulk species. We assume that electrons interact neither in entropic nor in elastic manner with the other surface species and thus only contribute to the reference energy. This assumption allows to consider the specific volume of the electrons to be vanishing.

Let $a_{M}^{\mathrm{ref}}$ and $a_{\alpha }^{\mathrm{ref}}$ denote the specific area of a metal ion and adsorbates of species $\alpha \in I_{S}$, respectively. We assume that the surface is built from an one atomic layer of metal ions that offers adsorption sites to the electrolyte species and the reaction products. Since some of the sites may be empty, we introduce the surface number density of vacancies by

(120) $$a_{V}^{\mathrm{ref}}\underset{s}{n}\;{\;}_{V}=a_{M}^{\mathrm{ref}}\underset{s}{n}\;{\;}_{M}-\;\;\;\sum_{\alpha \in I_{S}\backslash \left\{e,M\right\}}\;\;\;a_{\alpha }^{\mathrm{ref}}\underset{s}{n}\;{\;}_{\alpha }\;,$$

where $a_{V}^{\mathrm{ref}}$ is the specific area of the vacancies. Due to the assumptions, the electrons do not contribute to the surface coverage. For the formulation of the free energy contributions, it is useful to introduce total mole densities $\underset{s}{n}\;\;$ of adsorbates and the surface fractions $\underset{s}{y}\;{\;}_{\alpha }$ of adsorbates and vacancies, respectively,

(121) $$\underset{s}{n}\;\;=\;\;\;\sum_{\alpha \in I_{S}\backslash \left\{e,M\right\}}\underset{s}{n}\;{\;}_{\alpha }+\underset{s}{n}\;{\;}_{V}\;\;\mathrm{and}\;\;\underset{s}{y}\;{\;}_{\alpha }=\frac{\underset{s}{n}\;{\;}_{\alpha }}{\underset{s}{n}\;\;}\;\;\mathrm{with}\;\;\sum_{\alpha \in I_{S}\backslash \left\{e,M\right\}}\underset{s}{y}\;{\;}_{\alpha }+\underset{s}{y}\;{\;}_{V}=1\;.$$

The entropic contribution to the surface free energy is determined by statistical thermodynamics as in the bulk and takes into account the mixing entropy of the adsorbates and reaction products,

(122) $$\underset{s}{\rho }\;\;\underset{s}{\psi }\;{\;}^{\mathrm{mix}}=k_{B}T\sum_{\alpha \in I_{S}\backslash \left\{e,M\right\}}\left(\underset{s}{n}\;{\;}_{\alpha }\ln\left(\underset{s}{y}\;{\;}_{\alpha }\right)-\underset{s}{n}\;{\;}_{V}\ln\left(\underset{s}{y}\;{\;}_{V}\right)\right)\;.$$

The mechanical contribution of the free surface energy is based on a simple constitutive model for the surface tension. Let $\underset{s}{\gamma }\;{\;}^{\mathrm{ref}}$ be a reference surface tension and $\underset{s}{K}\;\;$ the surface modulus. Then, we set

(123) $$\underset{s}{\gamma }\;\;=\underset{s}{\gamma }\;{\;}^{\mathrm{ref}}+\underset{s}{K}\;\;\left(a_{M}^{\mathrm{ref}}\underset{s}{n}\;{\;}_{M}-1\right)\;.$$

Here, the surface tension is assumed to be a function of the metal ion density $\underset{s}{n}\;{\;}_{M}$ only. However, in general, adsorption and surface reaction may change the metal density and therefore the surface tension. Since in general $\underset{s}{K}\;\;$ is large for metals, small changes in $\underset{s}{n}\;{\;}_{M}$ lead to significant changes in the surface tension. Thus, the surface tension is also indirectly related to the surface coverage with adsorbates. Insertion of the definition (123) into constitutive equation (78)${}_{\mathrm{right}}$ and integration after an appropriate variable transformation, see [18], Appendix A, yields for the mechanical contribution to the free energy,

(124) $$\underset{s}{\rho }\;\;\underset{s}{\psi }\;{\;}^{\mathrm{mech}}=\underset{s}{\rho }\;\;\underset{s}{\psi }\;{\;}_{M}^{\mathrm{ref}}-\left(\underset{s}{\gamma }\;\;-\underset{s}{K}\;\;\right)\left(a_{M}^{\mathrm{ref}}\underset{s}{n}\;{\;}_{M}-1\right)-\underset{s}{K}\;\;\;a_{M}^{\mathrm{ref}}\underset{s}{n}\;{\;}_{M}\ln\left(a_{M}^{\mathrm{ref}}\underset{s}{n}\;{\;}_{M}\right)\;.$$

The resulting chemical potentials are

(125a) $$\underset{s}{\mu }\;{\;}_{\alpha }=\frac{1}{m_{\alpha }}\underset{s}{\psi }\;{\;}_{\alpha }^{\mathrm{ref}}+\frac{k_{B}T}{m_{\alpha }}\ln\left(\underset{s}{y}\;{\;}_{\alpha }\right)-\frac{k_{B}T}{m_{\alpha }}\frac{a_{\alpha }^{\mathrm{ref}}}{a_{V}^{\mathrm{ref}}}\ln\left(\underset{s}{y}\;{\;}_{V}\right)\;,\;\;\alpha \in I_{S}\backslash \left\{e,M\right\}\;,$$

(125b) $$\underset{s}{\mu }\;{\;}_{M}=\frac{1}{m_{M}}\underset{s}{\psi }\;{\;}_{M}^{\mathrm{ref}}+\frac{k_{B}T}{m_{M}}\frac{a_{M}^{\mathrm{ref}}}{a_{V}^{\mathrm{ref}}}\ln\left(\underset{s}{y}\;{\;}_{V}\right)-\frac{a_{M}^{\mathrm{ref}}}{m_{\alpha }}\;\left(\underset{s}{\gamma }\;{\;}^{\mathrm{ref}}+\underset{s}{K}\;\;\ln\left(\frac{\underset{s}{\gamma }\;\;-\underset{s}{\gamma }\;{\;}^{\mathrm{ref}}}{\underset{s}{K}\;\;}+1\right)\right)\;,$$

(125c) $$\underset{s}{\mu }\;{\;}_{e}=\frac{1}{m_{e}}\underset{s}{\psi }\;{\;}_{e}^{\mathrm{ref}}.$$

In contrast to the bulk, the surface chemical potentials of the adsorbates are independent from the stress tensor, i.e., surface tension, but we have contributions from the vacancies on the underlying lattice.

#### The Incompressible Limit

For liquid electrolytes, in particular for aqueous electrolytes, we expect that the volume does not change significantly if the pressure is varied. We can incorporate this behavior by means of the asymptotic limit $K/p^{\mathrm{ref}}\to \infty$. In an analogous way, we consider on the surface the limit $\underset{s}{K}\;\;/\underset{s}{\gamma }\;{\;}^{\mathrm{ref}}\to \infty$. In the incompressible limit, the relations (112) and (123) cannot be used anymore to determine the pressure *p* and the surface tension $\underset{s}{\gamma }\;\;$, respectively. Therefore, *p* and $\underset{s}{\gamma }\;\;$ become new independent unknowns of the system and, instead of the constitutive equations (112) and (123), there are the so-called incompressibility constraints

(126a) $$K/p^{\mathrm{ref}}\to \infty :\sum_{\alpha \in I}v_{\alpha }^{\mathrm{ref}}n_{\alpha }=1\;,$$

(126b) $$\underset{s}{K}\;\;/\underset{s}{\gamma }\;{\;}^{\mathrm{ref}}\to \infty :a_{M}^{\mathrm{ref}}\underset{s}{n}\;{\;}_{M}=1\;.$$

The chemical potentials become linear in the pressure respective surface tension, i.e.,

(127a) $$K/p^{\mathrm{ref}}\to \infty :\mu_{\alpha }=\frac{1}{m_{\alpha }}\psi_{\alpha }^{\mathrm{ref}}+\frac{v_{\alpha }^{\mathrm{ref}}}{m_{\alpha }}p+\frac{k_{B}T}{m_{\alpha }}\ln y_{\alpha }\;.$$

(127b) $$\underset{s}{K}\;\;/\underset{s}{\gamma }\;{\;}^{\mathrm{ref}}\to \infty :\underset{s}{\mu }\;{\;}_{M}=\frac{1}{m_{M}}\underset{s}{\psi }\;{\;}_{M}^{\mathrm{ref}}+\frac{k_{B}T}{m_{\alpha }}\frac{a_{M}^{\mathrm{ref}}}{a_{V}^{\mathrm{ref}}}\ln\underset{s}{y}\;{\;}_{V}-\frac{a_{M}^{\mathrm{ref}}}{m_{\alpha }}\underset{s}{\gamma }\;\;\;.$$

In case of a constant chemical potential $\underset{s}{\mu }\;{\;}_{M}$, we get a relation between surface tension and mole fractions of vacancies $\underset{s}{y}\;{\;}_{V}$. We can thus get an explicit dependence of the chemical potentials of adsorbates on the surface tension by replacing the mole fraction $\underset{s}{y}\;{\;}_{V}$ by the surface tension $\underset{s}{\gamma }\;\;$.

#### Solvation

**Figure 2:**
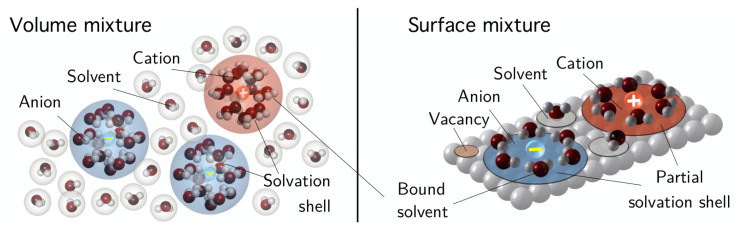
Sketch of the mixture constituents in the volume and on the surface. Anions and cations consist of a center ion and a surrounding solvation shell of bounded solvent molecules. In addition, there are the remaining free solvent molecules and on the surface there may be unoccupied sites (reprinted from [19], Figure 3, with permission from Cambridge University Press).

In polar solvents, in particular in water, the microscopic dipoles of the solvent molecules give rise to a microscopic electrostatic interaction between the solvent and the ionic species. This interaction leads to clustering of a finite number of solvent molecules around an ion, which is known as solvation. In Figure 2, the solvation of ions is illustrated. The solvation concept can be easily transferred from the volume to the surface as also illustrated in Figure 2. Solvation has a profound impact on the mixing entropy and the specific volume of the ions within the electrolyte model: solvent molecules that are bounded by the ions do not participate in the entropic interaction with the other constituents of the electrolytic mixture. Therefore, we handle an ion and its solvation shell as a single entity called solvated ion. Binding of solvent molecules into solvated ions decreases the amount of remaining free solvent molecules in the electrolyte.

Let $v_{0}^{\mathrm{ref}}$ denote the specific volume of a solvent molecule. The partial specific volume $v_{\alpha }^{\mathrm{ref}}$ of a solvated ion is greater than $v_{0}^{\mathrm{ref}}$ as well as it is considerably greater than the specific volume of un-solvated ions. We further assume for simplicity that the specific volume of the un-solvated ions is equal to $v_{0}^{\mathrm{ref}}$ and apply the very simple approximation $v_{\alpha }^{\mathrm{ref}}=\left(\kappa_{\alpha }+1\right)v_{0}^{\mathrm{ref}}$. Here, $\kappa_{\alpha }$ is the number of solvent molecules bounded by the ion and $\kappa_{\alpha }$ is called the *solvation number*.

### 7.3. The Electrochemical Double Layer in Equilibrium

At the electrode-electrolyte interface, ionic as well as electronic species can accumulate, forming boundary layers of only few nanometer thickness on both sides of the surface. This structure is commonly known as electric *double layer* [10,49]. It is a key feature of many electrochemical applications. For the theory presented here, the double layer is of particular interest because it allows valuable insight into the free energy which is not directly accessible to measurements. Since the width of the double layer is often much smaller than the macroscopic length scales of electrochemical components or cells, it can be asymptotically approximated by planar or radially symmetric (semi-)infinite systems.

#### Boundary Layer Structure

We consider a half space problem, where the domain x>0 is occupied by an incompressible liquid electrolyte. The region close to the boundary surface at x=0 represents the boundary layer. A potential difference is prescribed between x=0 and x→∞. From the constitutive relations derived in Section 6.1, we deduce that the equilibrium equations in the bulk resulting from the mass and momentum balance are, cf. [14],

(128a) $$\nabla (m_{\alpha }\mu_{\alpha }+z_{\alpha }e_{0}\varphi )=0\;\;\mathrm{for}\;\alpha \in I\;,$$

(128b) $$-\left(1+\chi \right)\varepsilon_{0}\Delta \varphi =n^{F}\;.$$

Integration of Equation (128a) and using the constitutive functions (127a) for the chemical potentials provides an implicit representation of the mole fractions, viz.

(129) $$y_{\alpha }=y_{\alpha }^{\infty }\exp\left(-z_{\alpha }\frac{e_{0}}{k_{B}T}\left(\varphi -\varphi^{\infty }\right)-\frac{v_{\alpha }^{\mathrm{ref}}}{k_{B}T}\left(p-p^{\infty }\right)\right)\;\;\mathrm{for}\;\alpha \in I\;,$$

where the mole fractions $y_{\alpha }^{\infty }$ and pressure $p^{\infty }$ at infinity are specified by bulk values of the electrolyte. Using the incompressibility constraint (126a), $n^{F}$ can be expressed as a function $n^{F}\left(\varphi ,p\right)$. For given boundary values of φ and *p*, the equilibrium state of the double layer is determined by the coupled system

(130) $$-\left(1+\chi \right)\varepsilon_{0}\Delta \varphi =n^{F}\left(\varphi ,p\right)\;,\sum_{\alpha \in I}y_{\alpha }\left(\varphi ,p\right)=1\;.$$

**Figure 3:**
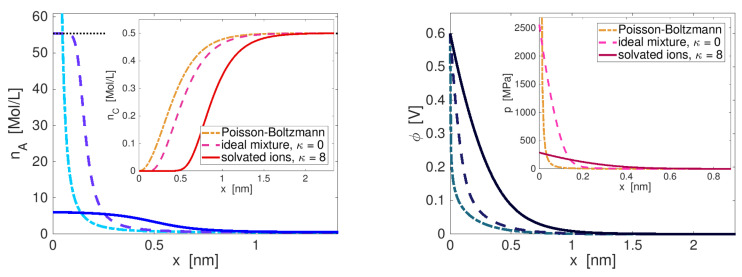
Comparison of models for an electrolyte with 0.5 Mol/L salt concentration in bulk and electrode potential 0.6 V larger than in bulk: standard Poisson–Boltzmann (dash-dotted line), ideal mixture of solvent and ions with equal specific volume (dashed line), and solvated ions with solvation number κ=8 (solid line). Pure solvent (water) has number density of 55.4 Mol/L (black pointed line); left: concentration profiles of anions (blue) and cations (red); right: profile of the potential (more blue colors) and corresponding pressure (more red colors).

The solution of the system (130) provides the spatial profiles of the ionic number densities in the double layer, cf. Figure 3. We observe that, in front of an electrode with a potential larger than in the electrolyte bulk, anions accumulate and for sufficiently large voltages the anion concentration saturates at a certain level that is related to the specific volume of the anions. Assuming that the anions have the same volume as the solvent, the saturation level is given by the number density of the pure solvent. In comparison, solvated ions are much larger then the pure solvent molecules and thus the saturation level is much lower and the saturated zone is much wider. Accordingly, the region where the cations are depleted is much wider for the solvated ions.

In contrast, classical Nernst–Planck/Poisson–Boltzmann theory is based on the assumption of a dilute solution and representations of the number densities as

(131) $$\text{Poisson-Boltzmann}:n_{\alpha }=n_{\alpha }^{\infty }\exp\left(-z_{\alpha }\frac{e_{0}}{k_{B}T}\left(\varphi -\varphi^{\infty }\right)\right)\;\;\mathrm{for}\;\alpha \in I\backslash \left\{0\right\}\;,$$

where 0 denotes the solvent. Compared to representation (129), here the dependence on the local pressure is missing and instead of the coupled system (130), only the single Poisson–Boltzmann equation $-\left(1+\chi \right)\varepsilon_{0}\Delta \varphi =n^{F}\left(\varphi \right)$ needs to be solved. However, Figure 3 demonstrates that already for moderate or even small applied voltages, classical Poisson–Boltzmann theory leads to unphysical, almost unlimited accumulation of ions, far beyond the number density of the pure solvent and thereby it violates the underlying strong dilution assumption. These limitations and inconsistencies of classical Poisson–Boltzmann theory are well known and we refer to [12] for a review of the extensions made in the literature.

Our approach based on non-equilibrium thermodynamics provides an additional major advantage over the classical theory and its extensions in the literature. From system (128) and the Gibbs–Duhem equation (78), we recover the momentum balance in equilibrium

(132) $$\nabla p=-n^{F}\nabla \varphi \;.$$

The Lorentz force $k=-n^{F}\nabla \varphi$ is balanced by the pressure gradient. Thus, pronounced pressure gradients have to be expected in charged boundary layers that screen the electric field. Figure 3 depicts the spacial profiles of potential and pressure in the boundary layer. We observe drastic increase of the pressure towards the electrode surface, where *p* grows up to several GPa. Although the solvated ion case shows considerably smaller pressure than the case of all particles having the same size, the pressure at the electrode is still orders of magnitude larger than atmospheric pressure of 0.1MPa. However, one has to keep in mind that the mechanical stress, which is controlled in experiments, and which can be measured, is the total stress (38). In the computations above, this total stress equals the outer pressure of 0.1MPa everywhere in space. Also for the classical Poisson–Boltzmann model, the local pressure can be calculated from (132). For the so computed pressure, we observe in Figure 3 a growth near the interface that is orders of magnitude stronger than in our model. In the one-dimensional setting we use here, a combination of Equations (128b) and (132) yields the relation $p-p^{\infty }=\frac{1+\chi }{2}\varepsilon_{0}{\left|\partial_{x}\varphi \right|}^{2}$. This reveals that in the Poisson–Boltzmann case, the almost unlimited accumulation of ions that shield the electric field at the surface leads to a steeper slope of the potential and thus to higher pressure, compared to our model with the solvated ions.

The electric charge of the boundary layer can be defined in the one-dimensional setting as

(133) $$Q_{\mathrm{BL}}=\int_{0}^{\infty }n^{F}\;dx\;.$$

A remarkable relation from ([18], Equation (150)) states

(134) $$Q_{\mathrm{BL}}=\mathrm{sgn}\left(\varphi \left(0\right)-\varphi^{\infty }\right)\sqrt{2\left(1+\chi \right)\varepsilon_{0}\left(p\left(0\right)-p^{\infty }\right)}\;.$$

This directly shows that classical Poisson–Boltzmann theory predicts the storage of an unrealistic huge charge in the boundary layer while our improved models considerably reduce the stored charge and the solvated ions approach can provide quantitatively meaningful results.

#### Double Layer Capacity

In addition to the charge accumulation in the double layer, also adsorption from the electrolyte to the surface and electron transfer reactions may take place, depending on the electrode metal and the ionic species. In a similar way like above, a surface charge $Q_{S}$ can be defined as function of the surface particle densities $\underset{s}{n}\;{\;}_{\alpha }$. The double layer charge is then $Q=Q_{\mathrm{BL}}+Q_{S}$.

In equilibrium, the solution of the boundary value problem for the double layer, in particular the double layer charge, becomes a function of the applied voltage *U* between electrode and electrolyte. This allows for the introduction of the *differential capacity* or *double layer-capacity* [9,10,49] as

(135) $$C=\frac{d}{dU}Q\left(U\right)\;.$$

As the charge *Q*, *C* also depends on the salt concentration, the temperature and the parameters of the free energy like specific volumes and adsorption energies.

**Figure 4:**
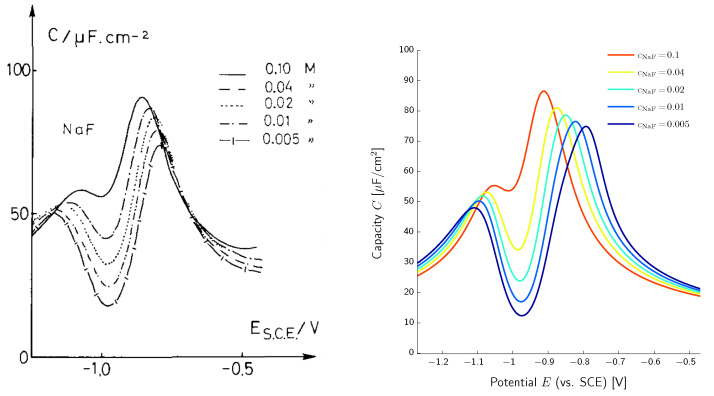
Left: measured capacity of the Ag(110)|NaF interface for salt concentrations in the range of (0.005–0.1) mol/L (reprinted from [50] with permission from Elsevier). Right: comparison of the computed capacity (reprinted from [18] with permission from Elsevier).

The double layer capacity can be measured directly and provides specific characteristics of the electrolyte and electrode–electrolyte interface at hand [50,51,52]. In Figure 4, measured and calculated double layer capacities are depicted for different salt concentrations. All simulations are done with the same set of parameters, independent from the salt concentration. For further details, we refer to [18]. The simulation shows the typical camel shape of the double layer capacity of aqueous electrolytes and a transition from a two-maximum curve for low salt concentrations to a one-maximum curve at high salt concentrations. Altogether, remarkable agreement between simulated and measured double layer capacities is reached.

#### Electrocapillarity—Lippmann Equation

Electrocapillarity describes the relationship between the interfacial tension γ and the applied voltage *U*. In a thin capillary tube, it can be observed that when a potential difference *U* is applied, a mercury–aqueous electrolyte interface moves according to the pressure difference while the curvature $k_{M}$ of the interface remains almost constant. The interfacial tension γ can be determined by the Young–Laplace equation,

(136) $$p^{+}-p^{-}=2k_{M}\;\gamma \;.$$

Moreover, the Lippmann equation [9,10,49]

(137) $$\frac{d}{dU}\gamma =-Q\;$$

relates the slope of the surface tension with respect to the applied voltage to the double layer charge.

When discussing electrocapillarity in the context of our model, some subtle differentiation is necessary. The interfacial tension γ in Equations (136) and (137) must not be identified with the surface tension $\underset{s}{\gamma }\;\;$ of Equations (78) or (123). In the context of non-equilibrium electrothermodynamics, the surface balance of momentum (22b) does not simplify to the Young–Laplace equation (136) due to the non-zero electromagnetic field.

In [19], we showed that in the asymptotic limit of thin double layers the Young–Laplace equation as well as the Lippmann equation can be derived. However, instead of the surface tension $\underset{s}{\gamma }\;\;$, these equations contain the newly defined interfacial tension

(138) $$\gamma =\underset{s}{\gamma }\;\;-\gamma_{\mathrm{BL}}^{+}-\gamma_{\mathrm{BL}}^{-}\;\mathrm{with}\;\gamma_{\mathrm{BL}}^{\pm }=\pm \int_{0}^{\pm \infty }\left(1+\chi \right)\varepsilon_{0}{\left|\partial_{r}\varphi \right|}^{2}\;dr\;.$$

**Figure 5:**
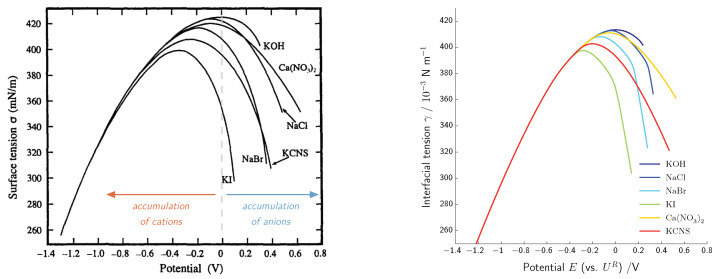
Left: measured electrocapillarity curves for various salts according to [53], Figure 1 (reprinted with permission from the American Chemical Society). Right: computed interfacial tension as described in [19], Figure 1 (reprinted with permission from Cambridge University Press).

Here, the two boundary layer contributions $\gamma_{\mathrm{BL}}^{\pm }$ of the corresponding electrode and electrolyte phase are non-negative functions of the electric field in the space charge layers. Thus, charging of the double layer always causes non-negative contributions $\gamma_{\mathrm{BL}}^{\pm }$ which lower the interfacial tension. This directly explains the U- or parabola-shaped electrocapillary curves which are observed in experiments—see Figure 5 for experimental measurements and comparison with simulation.

### 7.4. Electrochemical Systems in Non-Equilibrium

#### Generalized Nernst–Planck Flux

To derive an explicit representation of the partial mass fluxes, we substitute the chemical potentials (117) into the constitutive equations (59b). With the simplifying choice of a diagonal mobility matrix, where the diagonal elements are of the form $M_{\alpha \alpha }=M_{\alpha }\;T\;m_{\alpha }\rho_{\alpha }$, we get generalized Nernst–Planck fluxes. Denoting the solvent in Ω by the index 0, they read in the incompressible case for the ionic species α∈I\{0},

(139) $$J_{\alpha }=-M_{\alpha }\;m_{\alpha }k_{B}T\;\left(\nabla n_{\alpha }+n_{\alpha }\;\frac{z_{\alpha }e_{0}}{k_{B}T}\nabla \varphi \;-\frac{n_{\alpha }}{n_{0}}\frac{m_{\alpha }}{m_{0}}\nabla n_{0}\;-\frac{n_{\alpha }}{n}\left(1-\frac{m_{\alpha }}{m_{0}}\right)\nabla n\;+\frac{n_{\alpha }}{k_{B}T}\left(v_{\alpha }^{\mathrm{ref}}-\frac{m_{\alpha }}{m_{0}}v_{0}^{\mathrm{ref}}\right)\nabla p\right).$$

Compared to the standard Nernst–Planck model, cf. [9,10], there are three additional terms highlighted here in blue. The first of these three terms is due to the solvent–ion interaction. It originates from the construction of the entropy production (55) where we incorporated the constraint $\sum_{\alpha \in I}J_{\alpha }=0$. The second term results from the incompressibility constraint and the possibly different specific volume of the constituents. The third one contains the pressure contribution that is required for thermodynamically consistent fluxes. The pressure contribution only vanishes if the atomic masses and specific volumes of all species are equal. The classical Nernst–Planck equations are derived under the dilute solution assumption, cf. [54] and sometimes the additional assumption of locally electroneutral solution, cf. [55]. The validity of both assumptions cannot be guaranteed inside the double layer, as we have seen above. For a dilute solution, i.e., $n_{\alpha }\ll n_{0}$, the first two additional terms in the flux (139) can be neglected. Moreover, we will discuss next that, outside of the double layers, the electrolyte bulk can be considered locally electroneutral and in particular isobaric, causing also the third additional term in flux (139) to vanish. Thus, the generalized Nernst–Planck flux in a dilute electrolyte bulk reduces to the classical one. However, in the double layer, the difference in general is significant and even might dominate the overall behavior of the considered system.

#### Asymptotic Analysis and Reduced Models

**Figure 6:**
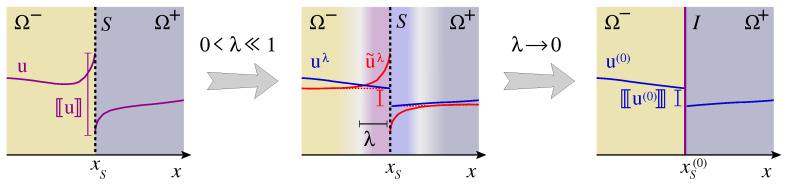
Generic field variable *u* for an electrochemical system of two substances, here metal and electrolyte, in contact with the surface S (**left**). Boundary layers due to a small parameter λ in the model equations and decomposition of *u* in bulk part $u^{\lambda }$ and boundary layer part ${\tilde{u}}^{\lambda }$ (**middle**). In a simplified model for λ→0, modified jump conditions contain the relevant information of the double layer that is not resolved any more (**right**). Figure reprinted from [16].

The partial mass balance equations with the fluxes (139) have to be coupled to the total mass and momentum balance, the Poisson equation and the surface (jump) conditions, leading to a rather complex system that contains several strongly different scales. In order to derive simpler models, formal asymptotic analysis can be applied, cf. [16,56] for a survey of the literature. An important characteristic parameter is λ according to definition (93). It relates the Debye length $\lambda^{2}x^{\mathrm{ref}}=\sqrt{\frac{k_{B}T\;\varepsilon_{0}}{e_{0}^{2}\;n^{\mathrm{ref}}}}$, that describes the width of the double layer, to characteristic macroscopic length $x^{\mathrm{ref}}$ of the system. For the asymptotic analysis, all dimensionless parameters are related to powers of λ. Then, any function is approximated by two different formal expansions with respect to λ, the *outer* expansion in the bulk and an *inner* expansion near the surface. For the inner expansion, the space coordinate is rescaled by λ. Figure 6 illustrates the asymptotic method. For each polynomial power in the parameter λ, the corresponding terms in the model equations are collected. Finally, both expansions are connected by matching conditions relating the boundary values of the outer expansion to the far field of the inner expansion. In [14,16,19], reduced models for the generalized Poisson–Nernst–Planck system are derived. They are characterized by following properties:

- In leading order, the bulk domain is locally electroneutral and pressure is constant in the bulk.
- The double layer is globally electroneutral and is in quasi-equilibrium. Thus, the results of the preceding section can be applied.
- Boundary layer charge and surface charge are both quantities of first higher order in λ.
- Analysis of the inner equations allows the formulation of new boundary conditions in terms of the outer variables.

#### Surface Reactions—Butler–Volmer Equation

Surface reactions, e.g., electron transfer reactions, have been intensely studied by experiments and there is a strong empirical basis for a macroscopic relation in which the surface reaction rate $\underset{s}{R}\;\;$ is driven by a potential difference at the interface that is called the *surface overpotential* $\eta_{S}$. This relation is known as the Butler–Volmer equation and is considered to be “the central equation in phenomenological electrode kinetics” ([49], p. 1053). It can be written as, cf. [9,10,49],

(140) $$\underset{s}{R}\;\;=R_{f}\;\exp\left(-\frac{\alpha_{f}\;e_{0}}{k_{B}T}\eta_{S}\right)\;-R_{b}\;\exp\left(+\frac{\alpha_{b}\;e_{0}}{k_{B}T}\eta_{S}\right)\;,$$

where $R_{f/b}$ are the forward and backward exchange rates, which may depend on the temperature *T* and bulk number densities $n_{\alpha }$. The transfer coefficients $\alpha_{f}$ and $\alpha_{b}$ are considered as constant phenomenological coefficients.

In non-equilibrium electrothermodynamics, we are confronted with the observation that the surface Maxwell’s equations (102) require the electric potential φ to be continuous at an interface, at least in the electrostatic setting. Therefore, no natural potential difference exists, which could be used to define an overpotential $\eta_{S}$.

By means of the asymptotic analysis above, it is possible to derive a general Butler–Volmer equation in the context of electrothermodynamics [17]. The derivation relies on two necessary conditions: (i) the boundary layers behave quasi-static such that the electrochemical potentials $\mu_{\alpha }+\frac{z_{\alpha }e_{0}}{m_{\alpha }}\varphi$ are constant and (ii) all adsorption processes are fast compared to the surface reactions, i.e., $\underset{s}{M}\;{\;}_{\alpha }\to \infty$ in the constitutive equations (72c).

Rather simple representations of the quantities defined in (140) in terms of the electrothermodynamic quantities can be found in the case of a single surface reaction where set of relevant surface constituents can be restricted to the constituents of the bulk phases, i.e., $I_{S}\backslash I=\emptyset$. In the thin double layer limit λ→0, we have

(141) $$\eta_{S}=-\left({\left(\varphi \right|}_{I}^{+}{-\varphi |}_{I}^{-})-(\bar{\varphi }{|}_{I}^{+}-\bar{\varphi }{|}_{I}^{-})\right)\;,$$

(142) $$R_{f}=\underset{s}{R}\;\;\;\exp\left(-\frac{\beta }{k_{B}T}\sum_{\alpha \in I}\underset{s}{\gamma }\;{\;}_{\alpha }m_{\alpha }\left(\mu_{\alpha }-{\bar{\mu }}_{\alpha }\right){|}_{I}^{\pm }\right)\;,$$

(143) $$R_{b}=\underset{s}{R}\;\;\;\exp\left(\frac{\left(1-\beta \right)}{k_{B}T}\sum_{\alpha \in I}\underset{s}{\gamma }\;{\;}_{\alpha }m_{\alpha }\left(\mu_{\alpha }-{\bar{\mu }}_{\alpha }\right){|}_{I}^{\pm }\right)\;.$$

In the asymptotic limit λ→0, we denote the thin double layer interface with its internal structure by *I*, in order to distinguish it from the physical surface *S* in the complete model (see Figure 6). The constant β∈(0,1) is known as the symmetry factor, which fosters either the forward or backward reaction. The transfer coefficients $\alpha_{f/b}$ depend on β and the stoichiometric coefficients $\underset{s}{\gamma }\;{\;}_{\alpha }$. The quantities supplied with an overbar represent equilibrium quantities that are defined by the Nernst equation $\sum_{\alpha \in I}\;\underset{s}{\gamma }\;{\;}_{\alpha }m_{\alpha }\left({\bar{\mu }}_{\alpha }+\frac{z_{\alpha }e_{0}}{m_{\alpha }}\bar{\varphi }\right){|}_{I}^{\pm }=0$.

In accordance with usual definitions in electrochemistry [10,49], the overpotential $\eta_{S}$ describes the deviation of the actual potential difference from the equilibrium voltage between the bulk phases. The exchange rates $R_{f/b}$ depend on temperature and in an indirect way via the bulk chemical potentials $\mu_{\alpha }$ on the bulk number densities.

To summarize: there exists a thermodynamic consistent basis for the Butler–Volmer equation and the thermodynamic origin is the constitutive relation (68) for arbitrary surface reactions. In contrast to the Butler–Volmer equation of electrokinetics, the general constitutive relation for surface reaction rates (68) can be applied to electrochemical systems where it is necessary to spatially resolve the electrical double layer and where rate limiting adsorption processes are involved. For a detailed derivation and analysis of general Butler–Volmer equations, we refer to [16,17].

## 8. Conclusions

Better theoretical understanding of many modern electrochemical applications demands extensions of classical continuum models. However, deriving such extensions in a thermodynamically consistent way is a non-trivial task, due to the coupled physical phenomena and the multi-scale nature of the considered systems. The derivation of the mathematical models greatly profits from the application of an entropy principle that restricts the modeling freedom. In the literature, several different variants or flavours of entropy principles can be found that often only differ in details. We chose an entropy principle oriented on [24] with the postulation of a specific structure of the entropy production as the sum of binary products. In this work, we focused on the coupling of electrodynamics and thermodynamics and the coupling of bulk and surface equations. We introduced classical balance equations for matter and Maxwell’s equations for the electromagnetic field with emphasis on the analogies between bulk and surface.

We restrict the constitutive modeling by a symmetry principle. Due to the different transformation properties of the (non-relativistic) balance equations of matter and Maxwell’s equations with the (1+3-dimensional) Maxwell–Lorentz aether relations for the electromagnetic field, the Galilean symmetry principle is chosen. By this choice, some relativistic effects are excluded, but typically these effects are negligible in electrochemical applications.

Another point one should draw attention to is the choice of independent variables for a specific considered material. This set of independent variables is not uniquely determined and different choices are possible. However, different choices imply different definitions of temperature, and they furthermore may lead to different stability properties, even though the entropy principle is satisfied, as we illustrated in Section 6.4. Thus, how can the appropriate set of independent variables be chosen? Typically, the choice is guided by experience and justified a posteriori by the derivation of the entropy production and the additional conditions for the relaxation to equilibrium.

Exploitation of the entropy principle reveals numerous cross relations between the derived constitutive equations. This is a consequence of having one pivotal ingredient to encode material behavior, i.e., the entropy density or the free energy density, respectively. In particular, bulk chemical potentials are defined as derivatives of the bulk entropy density and they appear e.g., in the bulk mass fluxes as well as in the adsorption conditions. Thus, these phenomena of a very different nature cannot be modeled independently.

How does the existing theory for charge transport in electrolytes and electrochemical processes at the electrode-electrolyte interfaces relate to the described general framework? As a first step to establish such relations, a dimensional analysis of general model is used to identify the relevant time and space scales for the application to electrolytes. This justifies substantial simplifications of the general model, in particular with respect to Maxwell’s equations. Next, an explicit model for the free energy density for liquid electrolytes is derived. The resulting liquid electrolyte model generalizes many of the already existing improved Nernst–Planck models with finite ion size effects such as e.g., [11,12]. Finally, by applying formal asymptotic analysis to the charged boundary layer at the electrode, it is possible to recover fundamental equations of electrochemistry like Butler–Volmer equations or the Lippmann equation.

While in principle it is possible to extend classical models by constitutive equations different from the ones derived here, it is in general not trivial to prove or disprove thermodynamic consistency of the obtained models. On the contrary, by applying the framework developed in this paper, the resulting models are guaranteed to be thermodynamically consistent. Moreover, starting from the more general framework, we gain deeper insights into the structure of electrochemical interfaces and the coupling of different phenomena.

### Outlook

Now that this very general modeling framework is developed, we are ready to apply it to many different electrochemical systems other than liquid electrolytes. To model e.g., solids and polymer electrolytes, appropriate material models have to be formulated in terms of a free energy density. In solids, one has to abstain from the simplifying assumption of the free energy being independent from the unimodular deformation gradient $F^{\mathrm{uni}}$ and one has to incorporate lattice velocities which generate further side conditions on the mass fluxes. For polymers, one has to incorporate the chain length of the polymers into the entropy of mixing and their impact on the elasticity.

With the introduction of the Galilean symmetry principle, the 1+3-dimensional Maxwell–Lorentz aether relations cannot be satisfied, but Lorentz invariance would be needed instead. The remedy is the consequent application of a relativistic setting for the equations of matter.

## Appendix A. Formal Solution of Surface Balances

Let *I* be some time interval and Ω an evolving domain that is intersected by the moving (singular) surface *S* into $\Omega^{+}$ and $\Omega^{-}$. By υ and $\underset{s}{\upsilon }\;\;$, we denote the barycentric velocity in the bulk and on the surface, respectively. Given the vector fields $F,G:I\times \Omega^{\pm }\to R^{3}$, define the density $n:I\times \Omega^{\pm }\to R$ and the flux $J:I\times \Omega^{\pm }\to R^{3}$ by

(A1) $$n=\mathrm{div}\left(F\right)\;\;\mathrm{and}\;\;J+n\upsilon =-\partial_{t}F+\mathrm{curl}\left(G\right)\;\mathrm{in}\;\Omega^{\pm }\;.$$

Moreover, assume that $\underset{s}{n}\;\;:I\times S\to R$ and the tangential surface flux $\underset{s}{J}\;\;:I\times S\to R^{3}$ satisfy the surface balance

(A2) $$\partial_{t,\nu }\underset{s}{n}\;\;+\left(\underset{s}{n}\;\;\underset{s}{\upsilon }\;{\;}_{\tau }^{\Delta }+\underset{s}{J}\;{\;}^{\Delta }\right){}_{∥\Delta }-2k_{M}\underset{s}{n}\;\;\underset{s}{\upsilon }\;{\;}_{\nu }=-\left[\;\left[J\cdot \nu +n\;\left(\upsilon_{\nu }-\underset{s}{\upsilon }\;{\;}_{\nu }\right)\right]\;\right]\;\mathrm{on}\;S\;.$$

Then, *n* is a conserved quantity under the flux J by construction and a formal solution of the surface balance (A2) is given by

(A3) $$\underset{s}{n}\;\;=\left[\;\left[F\cdot \nu \right]\;\right]\;\;\mathrm{and}\;\;\left(\underset{s}{n}\;\;\underset{s}{\upsilon }\;{\;}_{\tau }^{\Delta }+\underset{s}{J}\;{\;}^{\Delta }\right)\tau_{\Delta }=\nu \times \left[\;\left[G\right]\;\right]+\left[\;\left[F\right]\;\right]\underset{s}{\upsilon }\;{\;}_{\nu }-\underset{s}{n}\;\;\underset{s}{\upsilon }\;{\;}_{\nu }\nu .$$

To show the assertion, three identities are derived from (A3): First, (A3)${}_{1}$ is differentiated with respect to time *t*. With the definition (4), we infer

(A4) $$\begin{array}{lll} \partial_{t,\nu }\underset{s}{n}\;\;+\underset{s}{n}\;{\;}_{∥\Delta }\underset{s}{\upsilon }\;{\;}_{\tau }^{\Delta } & = & \left[\;\left[\partial_{t}F^{i}\nu_{i}+\partial_{m}F^{i}\underset{s}{\upsilon }\;{\;}^{m}\nu_{i}-F^{i}\left(\underset{s}{\upsilon }\;{\;}_{\nu ;\Gamma }+\underset{s}{\upsilon }\;{\;}_{\tau }^{\Sigma }b_{\Sigma \Gamma }\right)g^{\Gamma \Delta }\tau_{\Delta }^{i}\right]\;\right]\\ & \overset{\left(A1\right)}{=} & -\left[\;\left[J_{\nu }+n\upsilon_{\nu }\right]\;\right]+\left[\;\left[{\mathrm{curl}}_{i}G\right]\;\right]\nu^{i}+\left[\;\left[\partial_{m}F^{i}\right]\;\right]\underset{s}{\upsilon }\;{\;}^{m}\nu_{i}\\ & & \;-\left[\;\left[F^{i}\right]\;\right]\underset{s}{\upsilon }\;{\;}_{\nu ∥\Gamma }g^{\Gamma \Delta }\tau_{\Delta }^{i}-\left[\;\left[F^{i}\right]\;\right]\underset{s}{\upsilon }\;{\;}_{\tau }^{\Sigma }b_{\Sigma \Gamma }g^{\Gamma \Delta }\tau_{\Delta }^{i}. \end{array}$$

For the second identity, we define

(A5) $$\varepsilon^{ijk}=\left\{\begin{array}{cc} +1, & \mathrm{if}\;ijk\;\mathrm{is}\;\mathrm{an}\;\mathrm{even}\;\mathrm{permutation}\;\mathrm{of}\;123,\\ -1, & \mathrm{if}\;ijk\;\mathrm{is}\;\mathrm{an}\;\;\mathrm{odd}\;\mathrm{permutation}\;\mathrm{of}\;123,\\ 0, & \mathrm{else}. \end{array}\right.$$

Then, we differentiate (A3)${}_{2}$ with respect to the surface parameter $u^{\Sigma }$

(A6) $$\begin{array}{lll} & & -g^{\Sigma \Gamma }b_{\Sigma \Gamma }{\left(\tau_{\Sigma }\times \left[\;\left[G\right]\;\right]\right)}^{i}+\varepsilon^{ikl}\nu_{k}\left[\;\left[\partial_{m}G^{l}\tau_{\Sigma }^{m}\right]\;\right]+\left[\;\left[\partial_{m}F^{i}\tau_{\Sigma }^{m}\right]\;\right]\underset{s}{\upsilon }\;{\;}_{\nu }+\left[\;\left[F^{i}\right]\;\right]\underset{s}{\upsilon }\;{\;}_{\nu ∥\Sigma }\\ & = & \underset{s}{J}\;{\;}_{∥\Sigma }^{\Delta }\tau_{\Delta }^{i}+\underset{s}{J}\;{\;}^{\Delta }b_{\Delta \Sigma }\nu^{i}+{\left(\underset{s}{n}\;\;\underset{s}{\upsilon }\;{\;}_{\tau }^{\Delta }\right)}_{∥\Sigma }\tau_{\Delta }^{i}+{\left(\underset{s}{n}\;\;\underset{s}{\upsilon }\;{\;}_{\nu }\right)}_{∥\Sigma }\nu^{i}-\underset{s}{n}\;\;\underset{s}{\upsilon }\;{\;}_{\nu }g^{\Sigma \Gamma }b_{\Sigma \Gamma }\tau_{\Sigma }^{i}\\ \overset{\cdot \tau_{\Gamma }^{i}}{⟹} & & -g^{\Sigma \Gamma }b_{\Sigma \Gamma }\left[\;\left[G^{l}\right]\;\right]{\left(\tau_{\Sigma }\times \tau_{\Gamma }\right)}^{l}+\tau_{\Gamma }^{i}\varepsilon^{ikl}\nu^{k}\left[\;\left[\partial_{m}G^{l}\tau_{\Sigma }^{m}\right]\;\right]+\left[\;\left[\partial_{m}F^{i}\tau_{\Sigma }^{m}\tau_{\Gamma }^{i}\right]\;\right]\underset{s}{\upsilon }\;{\;}_{\nu }+\left[\;\left[F^{i}\tau_{\Gamma }^{i}\right]\;\right]\underset{s}{\upsilon }\;{\;}_{\nu ∥\Sigma }\\ & = & \underset{s}{J}\;{\;}_{∥\Sigma }^{\Delta }g_{\Delta \Gamma }+{\left(\underset{s}{n}\;\;\underset{s}{\upsilon }\;{\;}_{\tau }^{\Delta }\right)}_{∥\Sigma }g_{\Delta \Gamma }-\underset{s}{n}\;\;\underset{s}{\upsilon }\;{\;}_{\nu }g^{\Sigma \Gamma }b_{\Sigma \Gamma }g_{\Sigma \Gamma }\\ \overset{\cdot g^{\Gamma \Sigma }}{⟹} & & \varepsilon^{ikl}\nu^{k}g^{\Gamma \Sigma }\tau_{\Gamma }^{i}\left[\;\left[\partial_{m}G^{l}\tau_{\Sigma }^{m}\right]\;\right]+\left[\;\left[\partial_{m}F^{i}\tau_{\Sigma }^{m}\tau_{\Gamma }^{i}\right]\;\right]g^{\Gamma \Sigma }\underset{s}{\upsilon }\;{\;}_{\nu }+\left[\;\left[F^{i}\tau_{\Gamma }^{i}\right]\;\right]g^{\Gamma \Sigma }\underset{s}{\upsilon }\;{\;}_{\nu ∥\Sigma }\\ & = & \underset{s}{J}\;{\;}_{∥\Delta }^{\Delta }+{\left(\underset{s}{n}\;\;\underset{s}{\upsilon }\;{\;}_{\tau }^{\Delta }\right)}_{∥\Delta }-2k_{M}\underset{s}{n}\;\;\underset{s}{\upsilon }\;{\;}_{\nu }\\ ⟹ & & \varepsilon^{ikl}\nu^{k}\left(\delta^{im}-\nu^{i}\nu^{m}\right)\left[\;\left[\partial_{m}G^{l}\right]\;\right]+\left[\;\left[\partial_{m}F^{i}\right]\;\right]\left(\delta^{im}-\nu^{i}\nu^{m}\right)\underset{s}{\upsilon }\;{\;}_{\nu }+\left[\;\left[F^{i}\tau_{\Gamma }^{i}\right]\;\right]g^{\Gamma \Sigma }\underset{s}{\upsilon }\;{\;}_{\nu ∥\Sigma }\\ & = & \underset{s}{J}\;{\;}_{∥\Delta }^{\Delta }+{\left(\underset{s}{n}\;\;\underset{s}{\upsilon }\;{\;}_{\tau }^{\Delta }\right)}_{∥\Delta }-2k_{M}\underset{s}{n}\;\;\underset{s}{\upsilon }\;{\;}_{\nu }\\ \overset{\left(A1\right)}{⟹} & & -\left[\;\left[{\mathrm{curl}}_{k}G\right]\;\right]\nu^{k}+\left[\;\left[n\right]\;\right]\underset{s}{\upsilon }\;{\;}_{\nu }-\left[\;\left[\partial_{m}F^{i}\right]\;\right]\nu^{i}\nu^{m}\underset{s}{\upsilon }\;{\;}_{\nu }+\left[\;\left[F^{i}\right]\;\right]\tau_{\Gamma }^{i}g^{\Gamma \Sigma }\underset{s}{\upsilon }\;{\;}_{\nu ∥\Sigma }\\ & = & \underset{s}{J}\;{\;}_{∥\Delta }^{\Delta }+{\left(\underset{s}{n}\;\;\underset{s}{\upsilon }\;{\;}_{\tau }^{\Delta }\right)}_{∥\Delta }-2k_{M}\underset{s}{n}\;\;\underset{s}{\upsilon }\;{\;}_{\nu }. \end{array}$$

Finally, (A3)${}_{1}$ is differentiated with respect to the surface parameter $u^{\Delta }$,

(A7) $$\begin{array}{c} \left[\;\left[\partial_{m}F^{i}\right]\;\right]\tau_{\Delta }^{m}\nu^{i}-\left[\;\left[F^{i}g^{\Sigma \Gamma }b_{\Delta \Gamma }\tau_{\Sigma }^{i}\right]\;\right]=\underset{s}{n}\;{\;}_{∥\Delta }\\ \overset{\cdot \underset{s}{\upsilon }\;{\;}_{\tau }^{\Delta }}{⟹}\;\left[\;\left[\partial_{m}F^{i}\right]\;\right]\tau_{\Delta }^{m}\underset{s}{\upsilon }\;{\;}_{\tau }^{\Delta }\nu^{i}-\left[\;\left[F^{i}g^{\Sigma \Gamma }b_{\Delta \Gamma }\tau_{\Sigma }^{i}\right]\;\right]\underset{s}{\upsilon }\;{\;}_{\tau }^{\Delta }=\underset{s}{n}\;{\;}_{∥\Delta }\underset{s}{\upsilon }\;{\;}_{\tau }^{\Delta }. \end{array}$$

## Appendix B. Symmetry Properties of the Entropy Density

### Bulk

Let ρu+M·B, ${\left(\rho_{\alpha }\right)}_{\alpha \in I^{\pm }}$ be scalars, P, M vectors and $F^{\mathrm{uni}}$ a tensor with respect to Galilean transformation. Let *h* be an (absolute) scalar, i.e., *h* is invariant with respect to Galilean transformations according to Section 4. Moreover, let $\tilde{h}$ be a constitutive function such that

(A8) $$h=\tilde{h}\left(\rho u+M\cdot B,{\left(\rho_{\alpha }\right)}_{\alpha \in I^{\pm }},F^{\mathrm{uni}},P,M\right)\;.$$

Then, there holds the following symmetry relation for i,j=1,2,3

(A9) $$\frac{\partial \tilde{h}}{\partial F_{ik}^{\mathrm{uni}}}F_{jk}^{\mathrm{uni}}+\frac{\partial \tilde{h}}{\partial P^{i}}P^{j}+\frac{\partial \tilde{h}}{\partial M^{i}}M^{j}=\frac{\partial \tilde{h}}{\partial F_{jk}^{\mathrm{uni}}}F_{ik}^{\mathrm{uni}}+\frac{\partial \tilde{h}}{\partial P^{j}}P^{i}+\frac{\partial \tilde{h}}{\partial M^{j}}M^{i}\;.$$

To derive this relations, we use the transformation properties of all arguments of $\tilde{h}$ and the Galilean symmetry principle. Then, for any orthogonal matrix O, it follows

(A10) $$\begin{array}{ll} & \tilde{h}\left(\rho u+M\cdot B,{\left(\rho_{\alpha }\right)}_{\alpha \in I^{\pm }},F_{ik}^{\mathrm{uni}},P,M\right)\\ = & \;\tilde{h}\left(\rho u+M\cdot B,{\left(\rho_{\alpha }\right)}_{\alpha \in I^{\pm }},\det{\left(O\right)}^{-\frac{1}{3}}OF^{\mathrm{uni}},OP,\det\left(O\right)OM\right)\;. \end{array}$$

In particular, it is valid for rotations around the coordinate axes, which are characterized by the transformation matrices

(A11) $$R_{x}\;=\;\;\left(\begin{array}{ccc} 1 & 0 & 0\\ 0 & \cos\delta & -\sin\delta \\ 0 & \sin\delta & \cos\delta \end{array}\right)\;,R_{y}\;=\;\;\left(\begin{array}{ccc} \cos\beta & 0 & -\sin\beta \\ 0 & 1 & 0\\ \sin\beta & 0 & \cos\beta \end{array}\right)\;,R_{z}\;=\;\;\left(\begin{array}{ccc} \cos\gamma & -\sin\gamma & 0\\ \sin\gamma & \cos\gamma & 0\\ 0 & 0 & 1 \end{array}\right).$$

Here, δ,β and γ are the respective angles of rotation. Substituting $O=R_{x}$ into (A10) followed by differentiation with respect to δ yields

(A12) $$\frac{\partial \tilde{h}}{\partial F_{ik}^{\mathrm{uni}}}\frac{dR_{x}^{ij}}{d\delta }F_{jk}^{\mathrm{uni}}+\frac{\partial \tilde{h}}{\partial P^{i}}\frac{dR_{x}^{ij}}{d\delta }P^{j}+\frac{\partial \tilde{h}}{\partial M^{i}}\frac{dR_{x}^{ij}}{d\delta }M^{j}=0\;.$$

Then, we conclude for δ=0 that

(A13) $$\frac{\partial \tilde{h}}{\partial F_{2k}^{\mathrm{uni}}}F_{3k}^{\mathrm{uni}}+\frac{\partial \tilde{h}}{\partial P^{2}}P^{3}+\frac{\partial \tilde{h}}{\partial M^{2}}M^{3}=\frac{\partial \tilde{h}}{\partial F_{3k}^{\mathrm{uni}}}F_{2k}^{\mathrm{uni}}+\frac{\partial \tilde{h}}{\partial P^{3}}P^{2}+\frac{\partial \tilde{h}}{\partial M^{3}}M^{2}\;.$$

Applying the same arguments with the matrices $R_{y}$ and $R_{z}$ yields the assertion.

### Surface

Let $\underset{s}{\rho }\;\;\underset{s}{u}\;\;$, ${\left(\underset{s}{\rho }\;{\;}_{\alpha }\right)}_{\alpha \in I_{S}}$ be scalars and $\tau_{1/2}^{\mathrm{uni}}$ vectors with respect to Galilean transformation. Let $\underset{s}{h}\;\;$ be a scalar with respect to Galilean transformations. Moreover, let $\underset{s}{\tilde{h}}\;\;$ be a constitutive function

(A14) $$\underset{s}{h}\;\;=\underset{s}{\tilde{h}}\;\;\left(\underset{s}{\rho }\;\;\underset{s}{u}\;\;,{\left(\underset{s}{\rho }\;{\;}_{\alpha }\right)}_{\alpha \in I_{S}},\tau_{1}^{\mathrm{uni}},\tau_{2}^{\mathrm{uni}}\right)\;.$$

Then, the following symmetry properties hold:

(A15a) $$\frac{\partial \underset{s}{\tilde{h}}\;\;}{\partial \tau_{\Delta }^{\mathrm{uni},i}}\tau_{\Sigma }^{\mathrm{uni},i}g^{\Sigma \Gamma }=\frac{\partial \underset{s}{\tilde{h}}\;\;}{\partial \tau_{\Gamma }^{\mathrm{uni},i}}\tau_{\Sigma }^{\mathrm{uni},i}g^{\Sigma \Delta }\;\;\mathrm{for}\;\Gamma ,\Delta =1,2,$$

(A15b) $$\frac{\partial \underset{s}{\tilde{h}}\;\;}{\partial \tau_{\Delta }^{\mathrm{uni},i}}\nu^{i}=0\;\;\mathrm{for}\;\Delta =1,2.$$

Similar to the volume, the Galilean symmetry principle claim that, for any orthogonal matrix O, it holds

(A16) $$\underset{s}{\tilde{h}}\;\;\left(\underset{s}{\rho }\;\;\underset{s}{u}\;\;,{\left(\underset{s}{\rho }\;{\;}_{\alpha }\right)}_{\alpha \in I_{S}},\tau_{1}^{\mathrm{uni}},\tau_{2}^{\mathrm{uni}}\right)=\underset{s}{\tilde{h}}\;\;\left(\underset{s}{\rho }\;\;\underset{s}{u}\;\;,{\left(\underset{s}{\rho }\;{\;}_{\alpha }\right)}_{\alpha \in I_{S}},O\tau_{1}^{\mathrm{uni}},O\tau_{2}^{\mathrm{uni}}\right)\;.$$

Applying the same arguments as above for the rotations given by (A11), we get

(A17) $$\frac{\partial \underset{s}{\tilde{h}}\;\;}{\partial \tau_{\Delta }^{\mathrm{uni}}}\times \tau_{\Delta }^{\mathrm{uni}}=0\;.$$

Moreover, we easily conclude

(A18) $$g^{2\Delta }\tau_{\Delta }^{\mathrm{uni}}=\nu \times \tau_{1}^{\mathrm{uni}}\;\;\mathrm{and}\;\;g^{1\Delta }\tau_{\Delta }^{\mathrm{uni}}=-\nu \times \tau_{2}^{\mathrm{uni}}\;.$$

Multiplication of (A17) with ν and using (A18) yields (A15a) and (A15b) follows from (A17) after multiplication of (A17) by $\tau_{\Delta }^{\mathrm{uni}}$.
